# Mutational signatures in GATA3 transcription factor and its DNA binding domain that stimulate breast cancer and HDR syndrome

**DOI:** 10.1038/s41598-021-01832-z

**Published:** 2021-11-23

**Authors:** Atlal El-Assaad, Zaher Dawy, Athar Khalil, Georges Nemer

**Affiliations:** 1 Department of Electrical and Computer Engineering, American University of Beirut (AUB), Riad El Solh, Beirut, Lebanon; 2 Department of Biochemistry and Molecular Genetics, American University of Beirut (AUB), Riad El Solh, Beirut, Lebanon; 3 Department of Computer Science, Lebanese International University (LIU), Bekaa, Lebanon

**Keywords:** Cancer, Computational biology and bioinformatics, Drug discovery, Molecular biology, Structural biology, Biomarkers, Diseases, Medical research, Molecular medicine, Oncology, Engineering

## Abstract

Transcription factors (TFs) play important roles in many biochemical processes. Many human genetic disorders have been associated with mutations in the genes encoding these transcription factors, and so those mutations became targets for medications and drug design. In parallel, since many transcription factors act either as tumor suppressors or oncogenes, their mutations are mostly associated with cancer. In this perspective, we studied the *GATA3* transcription factor when bound to *DNA* in a crystal structure and assessed the effect of different mutations encountered in patients with different diseases and phenotypes. We generated all missense mutants of *GATA3* protein and DNA within the adjacent and the opposite *GATA3:DNA* complex models. We mutated every amino acid and studied the new binding of the complex after each mutation. Similarly, we did for every *DNA* base. We applied Poisson-Boltzmann electrostatic calculations feeding into free energy calculations. After analyzing our data, we identified amino acids and DNA bases keys for binding. Furthermore, we validated those findings against experimental genetic data. Our results are the first to propose in silico modeling for *GATA:DNA* bound complexes that could be used to score effects of missense mutations in other classes of transcription factors involved in common and genetic diseases.

## Introduction

The family of proteins that code for transcription factors is considered the largest family among all proteins types (about 10%). Specifically, 2600 proteins in the human genome contain *DNA*-binding domains, and most of them code for transcription factors^1^. The *GATA3* TF is encoded in humans by the *GATA3* gene and it controls the expression of a wide range of biologically and clinically important genes. *GATA3* belongs to the *GATA* family of zinc finger transcription factors, which are named according to their DNA binding subsequence ‘*GATA*’.

Many studies confirmed that *GATA3* mutations are involved in the development of certain types of breast cancer in humans^2^. *GATA3* was shown to be one of the three genes mutated in > 10% of breast cancers^3^. Some studies on mice indicated that *GATA3* is critical for the normal development of breast tissue and directly regulates luminal cell differentiation, whereas other studies indicated that it is integral to the expression of estrogen receptor alpha and to the signaling of androgen receptor^4–6^. Approximately one-half of the *GATA3* mutations identified in patients with breast cancer are clustered in exons 5 and 6, which encode *ZnF2* and the C terminal domain of the protein^7^. Experimental evidence showed that *ZnF2* of *GATA3* is required for *DNA* binding^8,9^. 15% of the mutations published in male breast cancer are present in *GATA3*, with hotspots recorded at residues S308 and S407 in luminal A and luminal B subtypes, respectively^10^.

Besides the different associations of *GATA3* with different forms of breast cancer, *GATA3* has been associated with hypoparathyroidism, deafness, and renal dysplasia (HDR) syndrome. The first described missense mutation (Leu348Arg) in HDR patients does not alter DNA binding or the affinity but likely alters the conformational change that occurs during binding in the *DNA* major groove^11^. Other mutations of *GATA3* TF, causing the HDR dysplasia syndrome, include: Two nonsense mutations Glu-228 to Stop and Arg-367 to Stop, one acceptor splice site mutation that leads to a frameshift from codon 351, a premature termination at codon 367, and two missense mutations: Cys-318 to Arg and Asn-320 to Lys. Mutations involving *GATA3 ZnF2* or adjacent basic amino acids result in a loss of *DNA* binding, but those involving *GATA3 ZnF1* either lead to a loss of interaction with FOG2 (Friend of *GATA*, cofactor) or alter *DNA*-binding affinity^12–18^.

In this work, we studied all possible amino acids and DNA bases missense mutations on two protein-*DNA* complexes (*GATA3:DNA* complex models with PDB ID: 3DFV and 3DFX). We applied Poisson–Boltzmann electrostatic study for the analysis of those mutations. The original method has been applied on many protein–protein complexes, and for *ionic*-only amino acids. In this context, we studied the role of *every* amino acid, and the role of *every DNA* base in regard to binding, and that is by mutating each amino acid and mutating each of Subsequence_1__*GATA*_Subsequence_2_ *DNA* bases (i.e., Subsequence_1__*GATA*_Subsequence_2_ represents the *DNA* sequence where the *GATA3* TF protein binds, and where Subsequence_1_ and Subsequence_2_ can be any of the four DNA bases: Adenine (A), Cytosine (C), Thymine (T), or Guanine (G)) that might lead to malfunction in transcription. Unlike previous studies, we hereby assessed the role of all amino acids (*ionic* and *non-ionic*) of the *GATA3* protein and the role of all *DNA* bases of the Subsequence_1__*GATA*_Subsequence_2_ *DNA* sequence during non-specific recognition (between amino acids and *DNA* backbone) and in specific binding (between amino acids and *DNA* bases).

This paper is organized as follows: We first described the computational method applied to the study of amino acids and *DNA* bases mutations in Section II. We then illustrated in details the results of applying the method in Section III; we revealed key amino acids and key *DNA* bases for complex binding, in addition to revealing the amino acids and *DNA* bases that play neutral roles in binding. Afterwards, we discussed those results, linked them to disease phenotypes, and validated them with published experimental data. Finally, we drew conclusions in Section IV and presented related future work.

## Methods

Recognition and binding form the two major steps of electrostatic association between protein and *DNA* molecules^19^. Nonspecific long-range electrostatic interactions characterize recognition, whereas specific favorable local short-range electrostatic interactions, such as hydrogen bonds, salt bridges, medium-range coulombic interactions, in addition to hydrophobic and van der Waals interactions, characterize binding. An accelerated weak encounter complex is formed during recognition. Contrariwise, the protein and *DNA* are locked into their final bound conformation during binding, and this occurs after local side change rearrangements, and exclusion of solvent atoms from their binding interface.

Several diseases were revealed through the effect of charged amino acids^20^. Similar diseases include the eye disease known as Age-related Macular Degeneration (AMD)^21^, the kidney disease known as atypical Hemolytic Uremic Syndrome (aHUS), the Dense Deposit Disease (DDD), also known as membranoproliferative glomerulonephritis^22^, and immune system disorder (over-activity or under-activity)^23^. The electrostatic type of interactions was shown in complexes like *C3d–CR2*^24–27^ and *C3d–EfbC/Ehp* association^26–29^, and in interactions with viral proteins *VCP/SPICE*^30,31^ and Kaposica^32^. The functional properties of each subunit of the E1 heterodimer activating-enzyme for *NEDD8*, *UBA3*, and *APPBP1* was studied electrostatically in^33^. Hierarchical clustering analysis of electrostatic potentials and charges of V3 loop of *HIV-1*, which plays a crucial role in viral entry into cells, was performed in^34^ and was mainly mediated by electrostatics. Single-alanine mutants of charged residues in the complexes *CD46(SCR1-2)-Ad11k* and *CD46(SCR1-2)-Ad21k* were computationally generated to mark out specific interfacial electrostatic interactions that are critical for association^35^.

*SUMO4*, a type 1 diabetes susceptibility gene, was found amenable to *SENP2*—a protease enzyme that processes *SUMO* into conjugatable form—processing via a single amino acid mutation through electrostatic computational modeling, and a combination of two amino acid mutations makes it highly accessible to *SENP2* substrate^36^. Electrostatic detailed investigation of factor H (*FH*) complement control protein (*CCP*) modules, in which mutations are linked to autoimmunity, revealed three binding sites in binding to complement protein *C3b*, thus increasing the affinity of *FH* for host surfaces^37^. Similar to *FH*, mutations in the MAC complex (*C5b6*) can lead to autoimmune diseases. Correspondingly, an electrostatic study of the interaction between *C5b* and *C6* complement proteins was completed in^38^. Electrostatic similarity methods applied to perturbed structures of *C3d* and *Cr2* revealed electrostatic “hot-spots” at the two functional sites of *C3d* and a lack of electrostatic “hot-spots” at the surface of *Cr2*, despite its excessively positive nature^39^. Additional electrostatic computational approaches were used to gain insight into the binding mode of the *C3d:CR2* complex^40^. Theoretical alanine-scanning mutagenesis and validation with experimental data was completed on five protein complexes in order to discern the role of individual ionized amino acids to protein association^41^.

### Protein-DNA interactions: framework description

Binding free energy calculations of many protein–protein interactions were implemented using the integrated Analysis of Electrostatic Similarities Of Proteins (AESOP) framework^26,27,29,33–42^. We based the electrostatic study of protein-DNA interactions on the same framework because protein-DNA interactions and protein–protein interactions share the same types of interactions; they both comprise bonded (bond, angle, torsion) and non-bonded (short-range and long-range electrostatic, van der Waals (vdW), and hydrogen bonds) interactions, as depicted in Table 1. ^43^ Intra-molecular represents interactions within the same molecule, whereas inter-molecular represents interactions between different molecules, like protein and DNA molecules. We then *expanded* AESOP to study all types of amino acids (*ionic* and *non-ionic*), in addition to all *DNA* bases and *DNA* backbone.

**Table 1:** Types of protein-DNA interactions. Non-bonded Specific refers to interactions between Amino Acid (AA) and *DNA* bases and Non-bonded Non-specific refers to interactions between AA and *DNA* backbone.

**Intra-molecular**			
Bonded	Bond	Angle	Torsion
Non-bonded	vdW	H-bond	Ionic
**Inter-molecular**			
Non-bonded specific	H-bond		
Non-bonded non-specific	vdW	H-bond	Ionic

The *Expanded-*AESOP framework encompasses the following steps:

#### Preparation of mutants from all GATA3 protein amino acids and from all nucleotides of the DNA sequences

R scripts were implemented to generate all types of mutants. We used as input the two forms of *GATA3:DNA* crystal structures (3DFV and 3DFX) from the Protein Data Bank (PDB). We replaced every amino acid—expected to be charged at physiological pH—one at a time, with each of the other nineteen amino acids. Along, we also replaced every base of the *DNA* sequence Subsequence_1__*GATA*_Subsequence_2_ with each of the other three *DNA* bases (e.g., G is mutated with A, C, or T).

#### Calculation of Poisson–Boltzmann electrostatic potentials

The electrostatic potentials were calculated using APBS software^43^, which is based on the linearized Poisson–Boltzmann equation, as illustrated in previous studies^25–27,29^. The atomic radii and charges, needed for APBS calculations, were calculated using PDB2PQR^44^ program and AMBER force field parameters^45,46^.

#### Calculation of electrostatic free energies of complex binding

The calculations of the electrostatic potentials were fed into the calculations of the electrostatic free energies of binding based on a thermodynamic cycle, as described in^26,27,29^, and in the form of the following equations:

1a $$\Delta \Delta G\begin{array}{*{20}c} {association} \\ {solvation} \\ \end{array} = \Delta G\begin{array}{*{20}c} {GATA:DNA} \\ {solvation} \\ \end{array} - \Delta G\begin{array}{*{20}c} {GATA} \\ {solvation} \\ \end{array} - \Delta G\begin{array}{*{20}c} {DNA} \\ {solvation} \\ \end{array}$$

1b $$\Delta \Delta G\begin{array}{*{20}c} {association} \\ {solvation} \\ \end{array} = \Delta G\begin{array}{*{20}c} {association} \\ {solution} \\ \end{array} - \Delta G\begin{array}{*{20}c} {association} \\ {reference} \\ \end{array}$$

2 $$\Delta G\begin{array}{*{20}c} X \\ {solvation} \\ \end{array} = G\begin{array}{*{20}c} X \\ {solution} \\ \end{array} - G\begin{array}{*{20}c} X \\ {reference} \\ \end{array}$$

3 $$\Delta G\begin{array}{*{20}c} {association} \\ Y \\ \end{array} = G\begin{array}{*{20}c} {GATA:DNA} \\ Y \\ \end{array} - G\begin{array}{*{20}c} {GATA} \\ Y \\ \end{array} - G\begin{array}{*{20}c} {DNA} \\ Y \\ \end{array}$$

where Eq. (1a) presents the binding free energy of the complex in solvent. Equation (1b) presents the binding free energy of the complex after eliminating artifacts. Equation (2) presents the energy of the solvent after subtracting artifacts. Equation (3) presents the energy of the complex after subtracting individual components. Looking backwards, Eqs. (2) and (3) feed into Eqs. (1a) and (1b), whereas Eq. (1a) presents the final form of the complex *GATA3:DNA* binding free energy calculation.

#### Data visualization

We used data visualization to help scientists understand the significance of data by placing it in a visual context, such as patterns, trends, and correlations that might go undetected in text-based forms. Swiss PDB Viewer and Chimera represent the visualization software we used^47,48^.

## Results and discussion

### Real data

**Figure 1:**
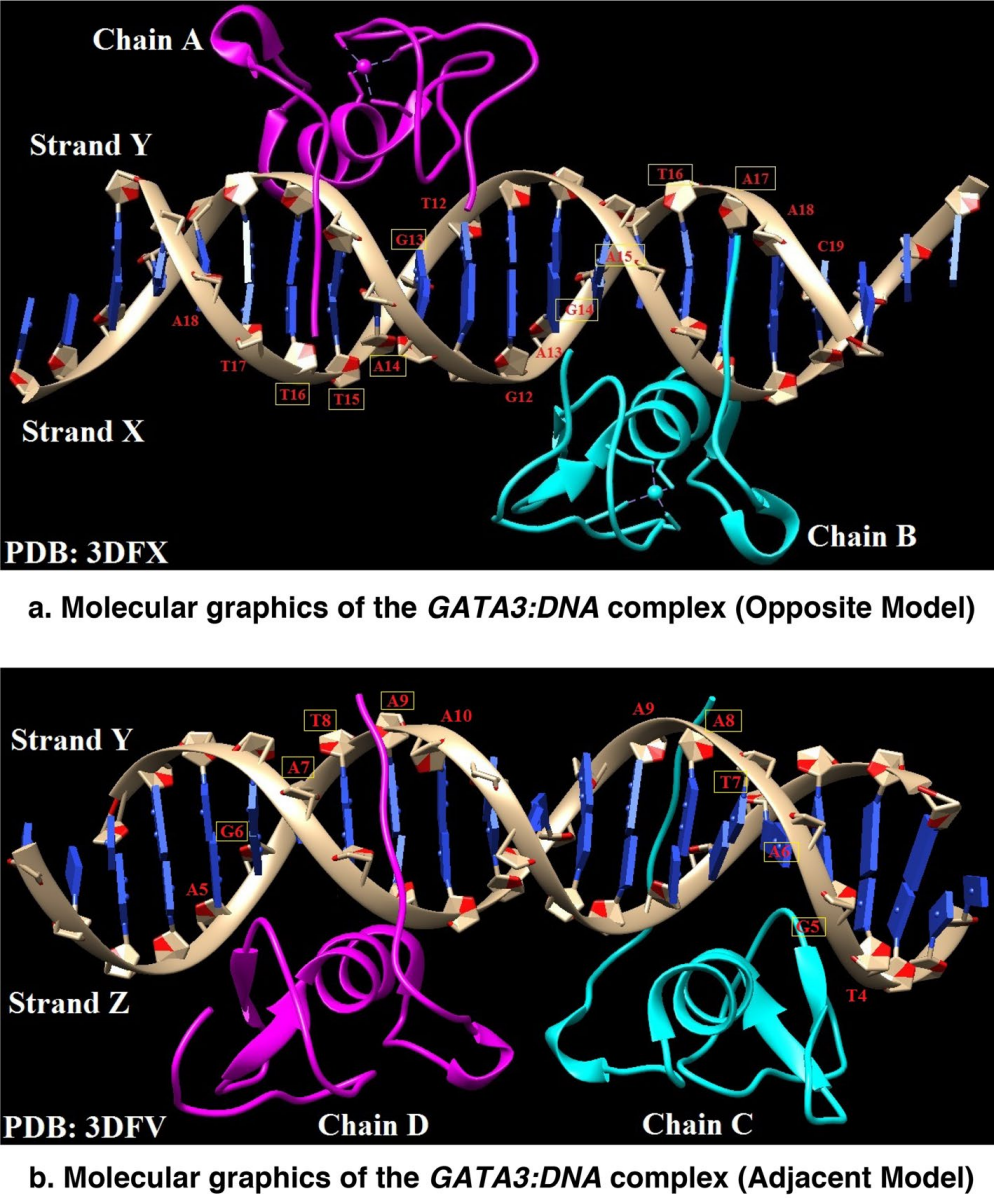
Molecular graphics of the *GATA3:DNA* complex. Analysis and visualization of the protein-*DNA* structure are performed using the visualization program Chimera^47^. In this *GATA3:DNA* complex, the net charge of *GATA3* is − 38e and that of *DNA* is 16e. (**a**) Plot presents the Opposite (OPP) model showing Chain-B interactions with the *DNA.* (**b**) Plot presents the Adjacent (ADJ) model showing Chain-C interactions with the *DNA*, in a similar fashion to Chain-B interactions with the *DNA* in the OPP model.

We studied the binding free energy calculations of *GATA3:DNA* complex (Fig. 1a, b). *GATA3:DNA* is available under two different conformations with PDB IDs: (a) 3DFX (*GATA3* binding to *DNA* in an opposite manner) and (b) 3DFV (*GATA3* binding to *DNA* in an adjacent manner)^16^.

The two crystal models share the same types of interactions. Accordingly, we used the available interactions of the Opposite model (OPP) to elaborate on the role of some amino acids hubs in the Adjacent model (ADJ). For electrostatic calculations, we used the crystal structure 3DFV, in which *GATA3* comprises the coordinates of amino acids Arg311-Arg366 for each of Chain-D and Chain-C, and in which the *DNA* module comprises the coordinates of nucleic acids from T1 to C20 for Strand-Y and from A1 to G20 for Strand-Z, as follows:

**DNA Strand-Z:** A_1_A_2_G_3_C_4_A_5_**G**_**6**_**A**_**7**_**T**_**8**_**A**_**9**_A_10_G_11_T_12_C_13_T_14_T_15_A_16_T_17_C_18_A_19_G_20_.

where

Subsequence_1_: AAGCA

Subsequence_2_: AGTCTTATCAG

**DNA Strand-Y:** C_20_G_19_T_18_C_17_T_16_A_15_T_14_T_13_C_12_A_11_G_10_A_9_**A**_**8**_**T**_**7**_**A**_**6**_**G**_**5**_T_4_C_3_T_2_T_1_.

where

Subsequence_1_: TTCT

Subsequence_2_: AGACTTATCTGC

As shown in Fig. 1b, Chain-D binds to ‘GATA’ subsequence on Strand-Z and Chain-C binds to ‘GATA’ subsequence (in reverse) on Strand-Y.

### Parameters

After some measurements and based on prior calculations, we set the parameters for *GATA3:DNA* calculations specifically as follows:

- The probe radii for defining the dielectric: 1.4 Å
- The probe radii of the ion accessibility surface: 2.0 Å
- The dielectric coefficient for the protein interior: 2
- The dielectric coefficient for the solvent: 78.54
- The grid used in the APBS calculations: 129 × 161 × 161 grid points
- The coarse grid lengths: 82 Å × 97 Å × 104 Å
- The fine grid lengths: 68 Å × 77 Å × 81 Å
- The grid resolution: ≤ 1 Å

### Mutational analysis from computational results

In order to detect the effect of each mutated perturbation on the overall binding ability of the complex *GATA3:DNA*, we performed the following steps: First, we generated a family of *GATA3* mutants and a family of *DNA* mutants from the crystallographic structure *GATA3:DNA*^43^ at atomic detail, and that is by replacing each amino acid with each of the other nineteen amino acids and replacing each base of the *DNA* sequence Subsequence_1__*GATA*_Subsequence_2_ with each of the other three *DNA* bases. Second, we performed Poisson–Boltzmann electrostatic calculations on each of those mutant complexes. Third, we performed electrostatic free energy calculations on each of those mutant complexes. Comparison of every mutant’s free energy calculation with the parent/wild *GATA3:DNA* complex free energy calculation, reveals the alteration effect of each mutated *GATA3* protein amino acid and of each mutated *DNA* base on binding, and subsequently, gives an indication of key amino acids and key *DNA* bases for binding.

The current dataset consists of one family of *GATA3* protein mutants and one family of *DNA* sequence mutants. Since each chain consists of 56 amino acids (numbered 311–366) and each amino acid is mutated to 19 other amino acids, we have a total of 1064 protein mutants per one chain of the 3DFV structure, or 2128 per both chains (Chain-C and Chain-D are symmetrical). The dataset also consists of a family of Subsequence_1__*GATA*_Subsequence_2_ *DNA* sequence mutants, where each *DNA* base is mutated to each of the three other *DNA* bases, composing a total of 60 mutants for each strand (Strand-Y and Strand-Z are complementary), or 120 *DNA* mutants per both strands.

**Figure 2:**
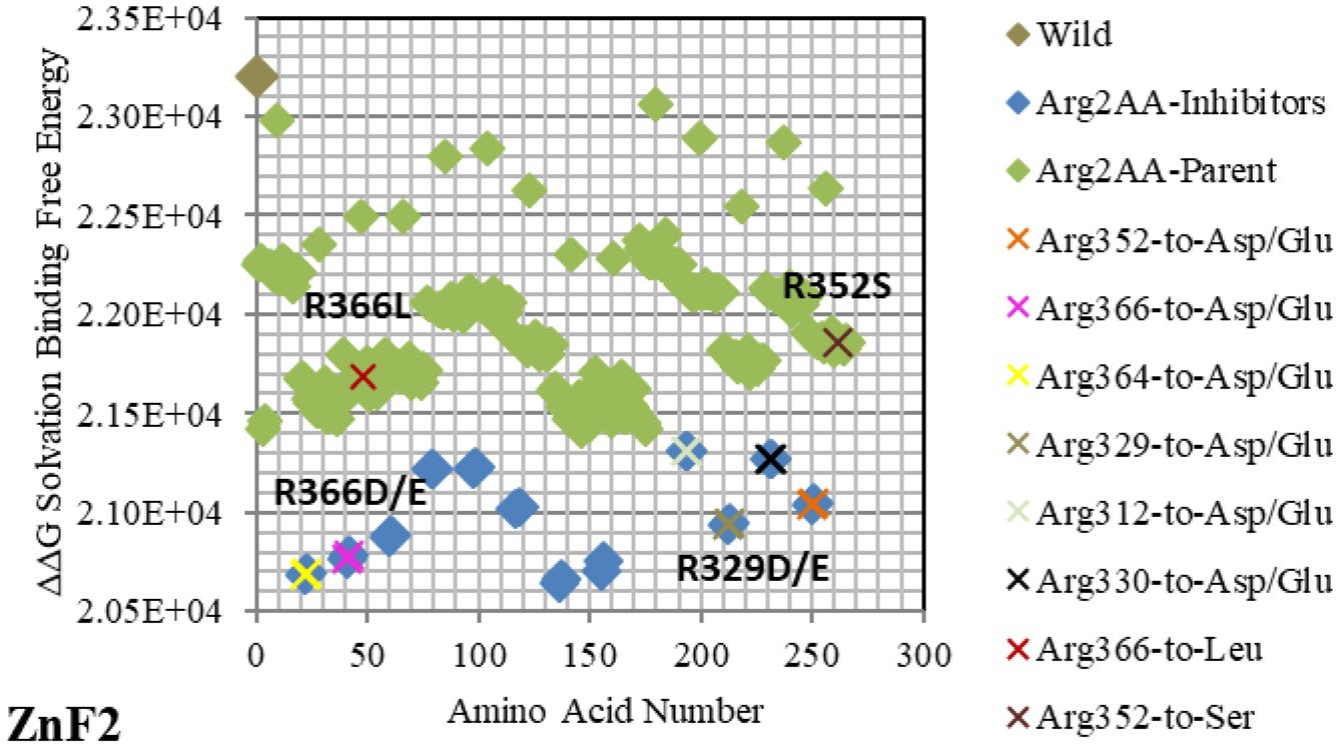
Electrostatic free energy differences of Arginine (Arg/R) within *GATA3:DNA* complex. Plot presents the solvated binding free energy calculations (in KJ/mol) of *GATA3* Arg amino acid mutants in both of Chain-D and Chain-C of the crystal structure 3DFV. Basic and acidic mutants are presented in blue and red colors respectively. Mutants shown above the parent/wild are predicted to enhance binding, whereas mutants shown below it are predicted to reduce binding. Due to its positive charge, Arg/R amino acid does not lead to acidic mutants. The x-axis (index) represents the order of the mutant (mutants are numbered and ordered sequentially). The amino acids shown with labels reflect the ones associated with diseases, whereas the amino acids presented with different colors of 'X' symbolize critical amino acids identified through the strong hubs of interactions they make.

**Figure 3:**
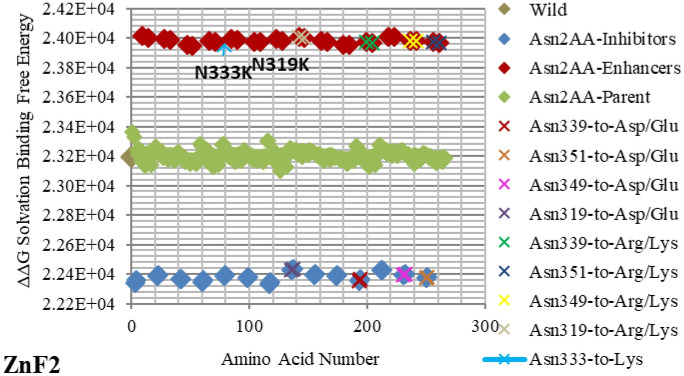
Electrostatic free energy differences of Asparagine (Asn/N) within *GATA3:DNA* complex. Plot presents the solvated binding free energy calculations (in KJ/mol) of *GATA3* Asn amino acid mutants in both of Chain-D and Chain-C.

**Figure 4:**
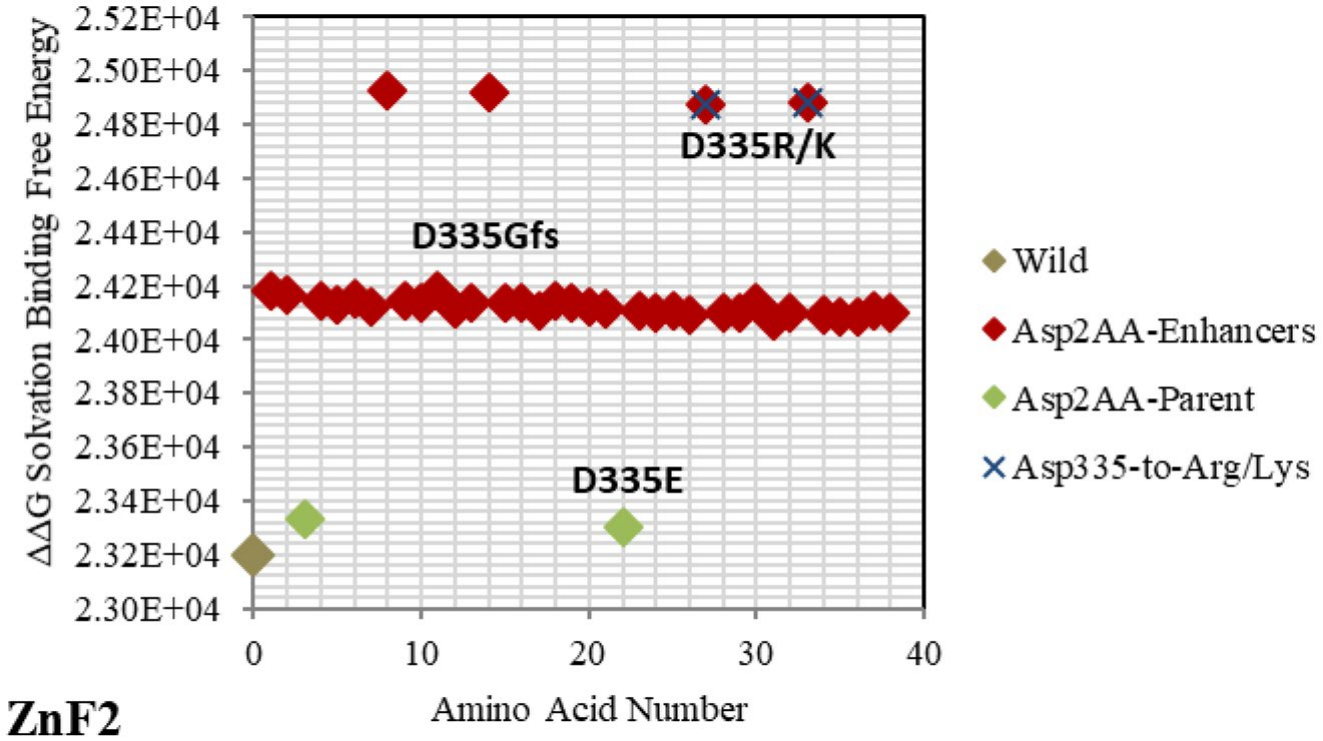
Electrostatic free energy differences of Aspartic Acid (Asp/D) within *GATA3:DNA* complex. Plot presents the solvated binding free energy calculations (in KJ/mol) of *GATA3* Asp amino acid mutants in both of Chain-D and Chain-C.

**Figure 5:**
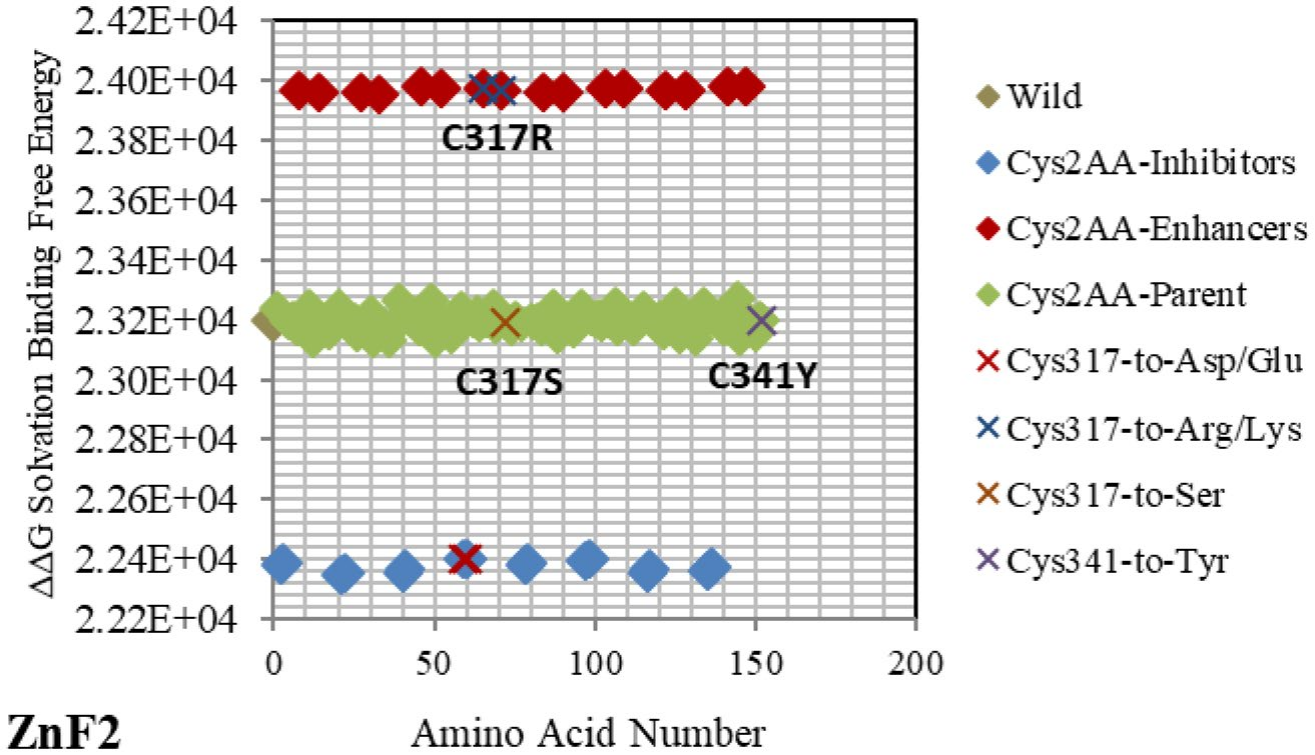
Electrostatic free energy differences of Cysteine (Cys/C) within *GATA3:DNA* complex. Plot presents the solvated binding free energy calculations (in KJ/mol) *GATA3* Cys amino acid mutants in both of Chain-D and Chain-C.

**Figure 6:**
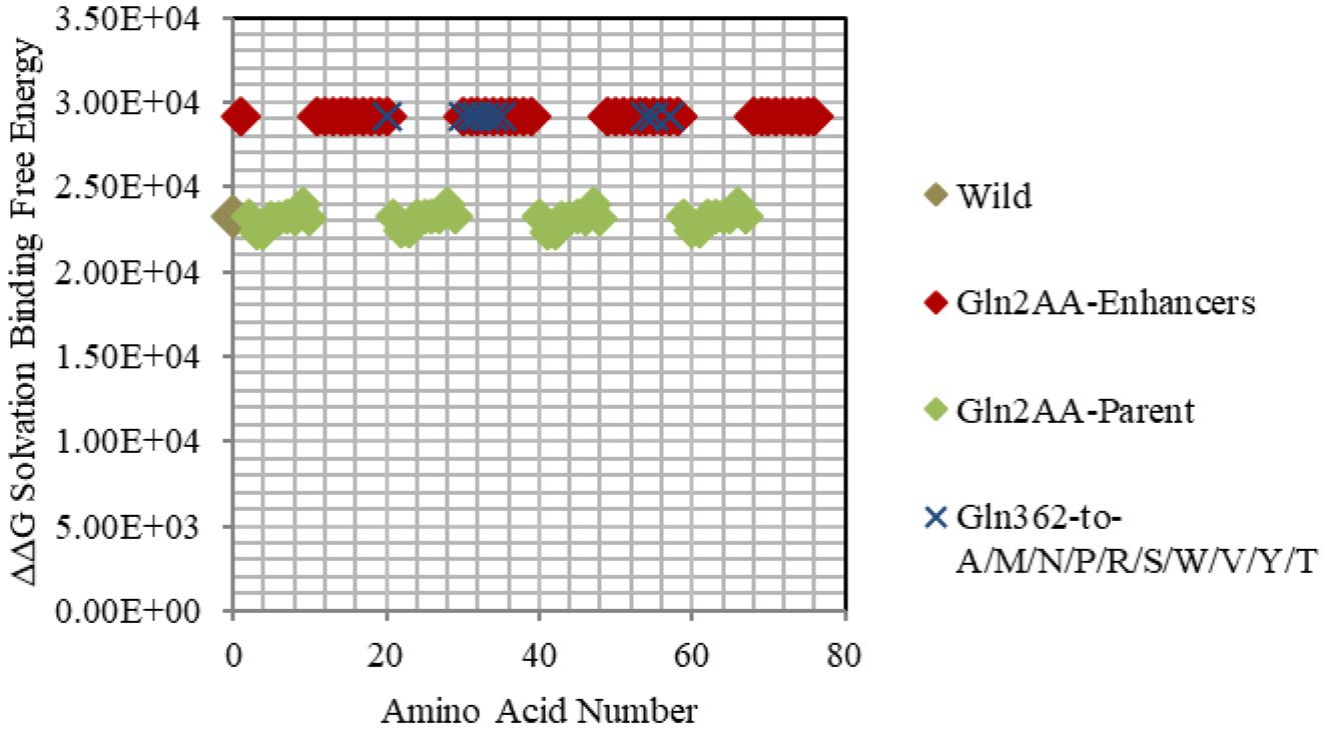
Electrostatic free energy differences of Glutamine (Gln/Q) within *GATA3:DNA* complex. Plot presents the solvated binding free energy calculations (in KJ/mol) *GATA3* Gln amino acid mutants in both of Chain-D and Chain-C.

**Figure 7:**
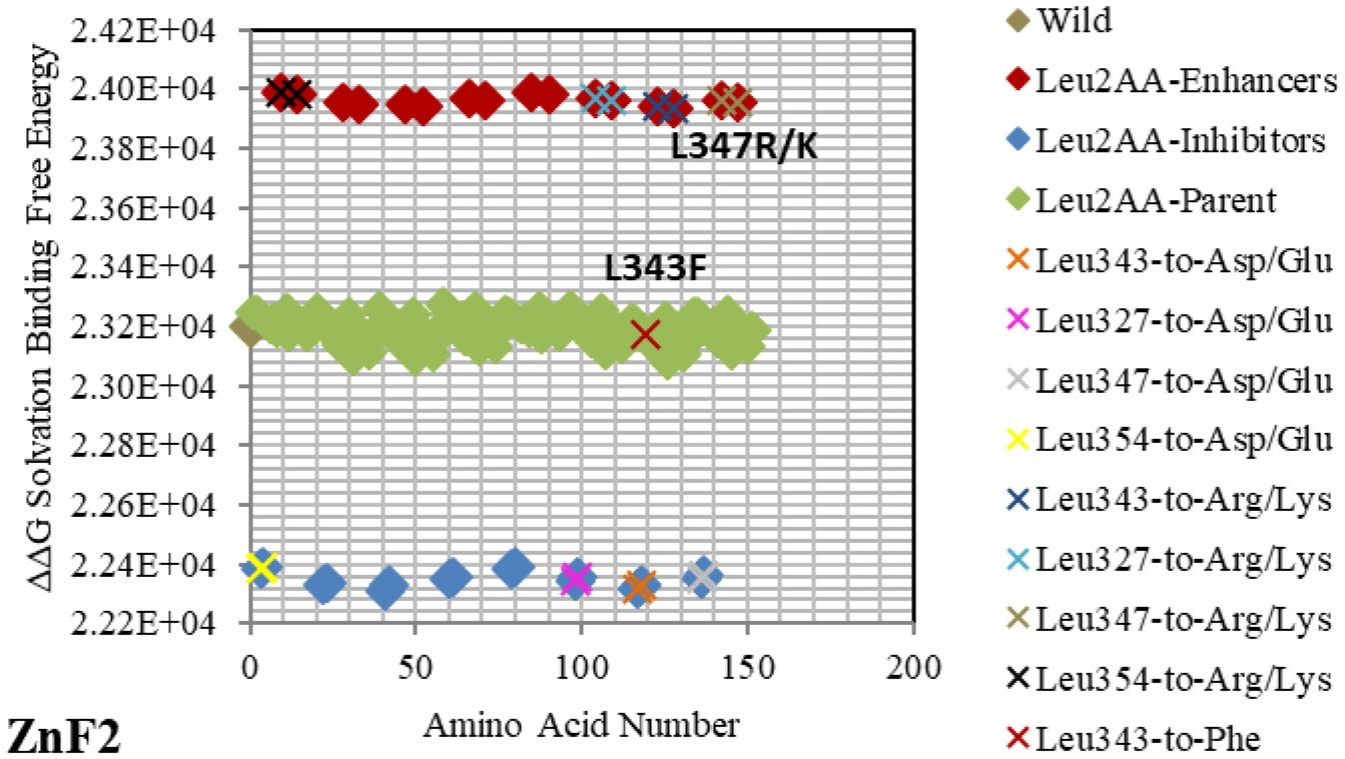
Electrostatic free energy differences of Leucine (Leu/L) within *GATA3:DNA* complex. Plot presents the solvated binding free energy calculations (in KJ/mol) of *GATA3* Leu amino acid mutants in both of Chain-D and Chain-C.

**Figure 8:**
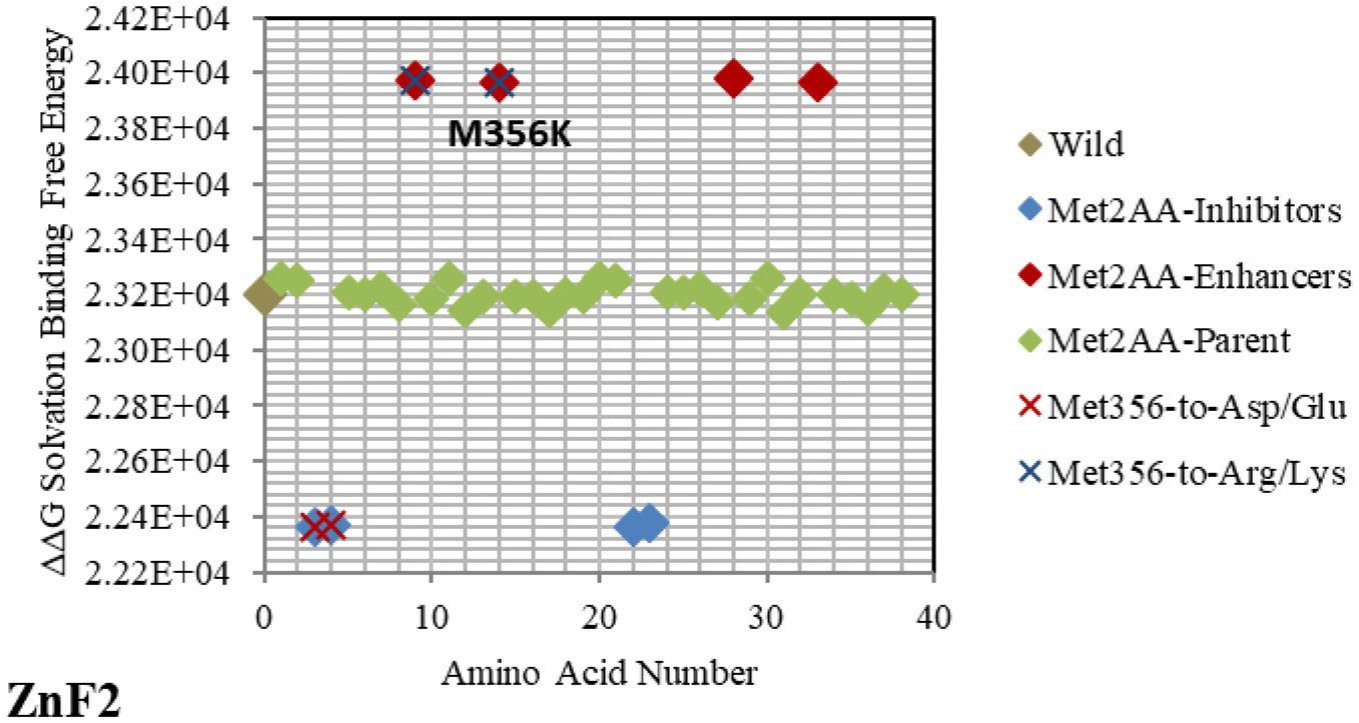
Electrostatic free energy differences of Methionine (Met/M) within *GATA3:DNA* complex. Plot presents the solvated binding free energy calculations (in KJ/mol) of *GATA3* Met amino acid mutants in both of Chain-D and Chain-C.

**Figure 9:**
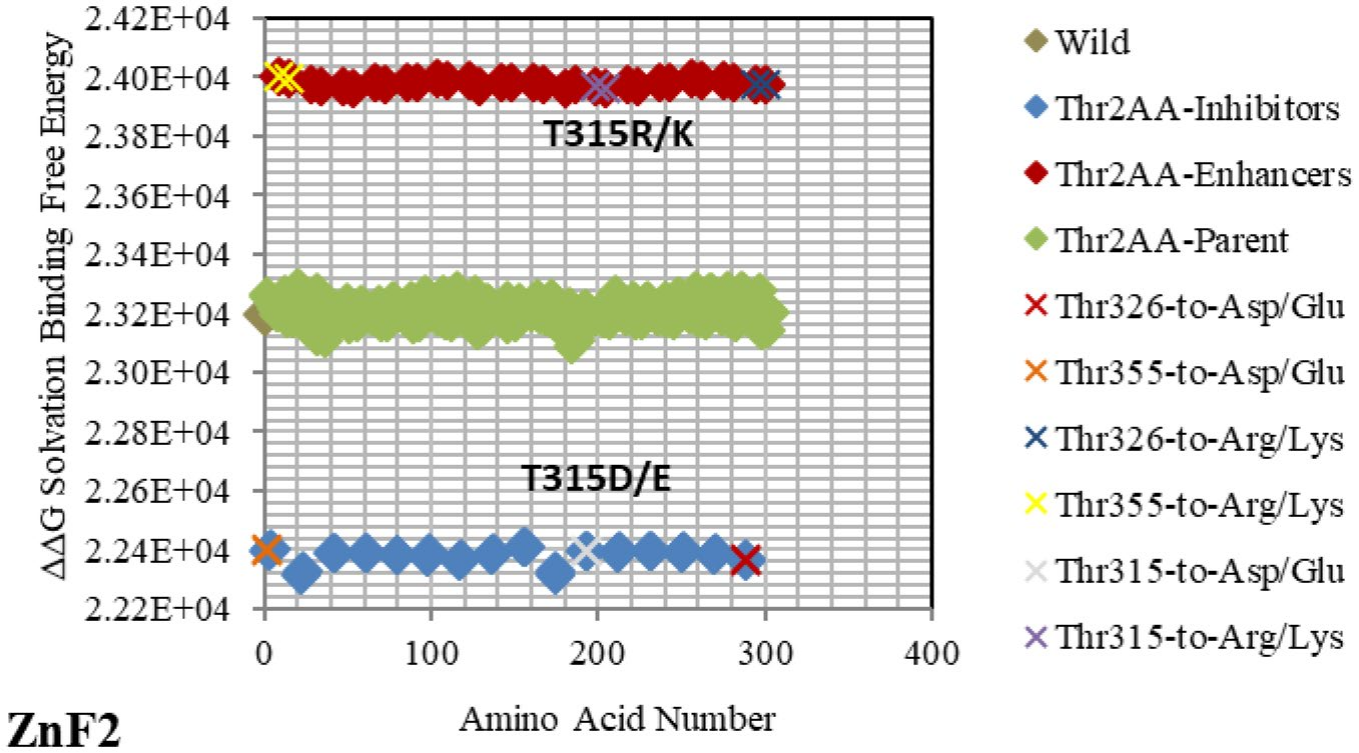
Electrostatic free energy differences of Threonine (Thr/T) within *GATA3:DNA* complex. Plot presents the solvated binding free energy calculations (in KJ/mol) of *GATA3* Thr amino acid mutants in both of Chain-D and Chain-C.

**Figure 10:**
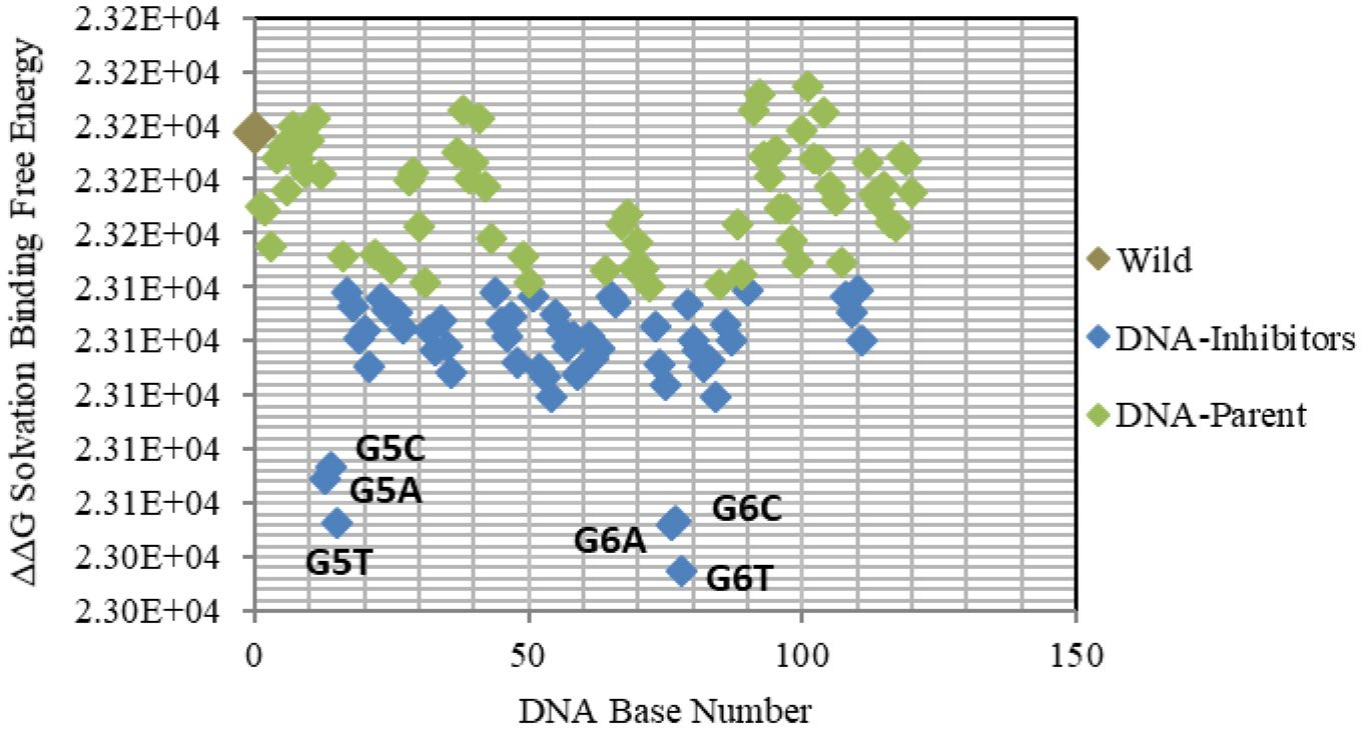
Electrostatic free energy differences of the *DNA* sequence within *GATA3:DNA* complex. Plot presents the solvated binding free energy calculations (in KJ/mol) of Subsequence_1__*GATA*_Subsequence_2_ mutations in Strand-Y and Strand-Z. Blue colors signify basic mutants. Those mutants are predicted to reduce binding since they are shown below the parent/wild. DNA bases illustrated with labels signify the strongest inhibitors.

We superimposed the structures of the *GATA3* protein mutants and those of the Subsequence_1__*GATA*_Subsequence_2_ *DNA* sequence using the backbone Cα atoms and centered them on the same grid used for the parent/wild structure (*GATA3:DNA*). Figures 2, 3, 4, 5, 6, 7, 8, 9 and 10 present the electrostatic free energy calculations at 150 mM ionic strength of major mutants of the complex *GATA3:DNA*; the corresponding tables Tables 2, 3, 4, 5, 6, 7, 8, 9 and 10 list the specific amino acids and *DNA* bases that play a key role in binding. All the rest of the mutants are detailed in Supplementary Figures SF1-SF11 and in Supplementary Tables ST1-ST11. The *solvation* free energy difference for each mutant is computed by Eq. (1a), and compared against the parent/wild protein-*DNA* complex solvation free energy. Such comparison serves as a physicochemical classifier of binding ability. The free energy difference of the difference ΔΔG is computed by Eq. (1b) based on the thermodynamic cycle described in section 2 of ^26,27,29^. For binding, an increase in *solvation* binding free energy ΔΔG is considered favorable, whereas a decrease is considered unfavorable.

**Table 2:** Arginine (Arg/A) amino acid mutants.

Inhibitor	
**R364D** (Arg #364 to Asp)	**R352D** (Arg #352 to Asp)
**R364E** (Arg #364 to Glu)	**R352E** (Arg #352 to Glu)
**R366D** (Ala #366 to Asp)	**R312D** (Arg #312 to Asp)
**R366E** (Ala #366 to Glu)	**R312E** (Arg #312 to Glu)
**R329D** (Ala #329 to Asp)	**R330D** (Arg #330 to Asp)
**R329E** (Ala #329 to Glu)	**R330E** (Arg #330 to Glu)

**Table 3:** Asparagine (Asn/N) amino acid mutants.

Enhancer	Inhibitor	Enhancer	Inhibitor
**N333R** (Asn #333 to Arg)	**N333D** (Asn #333 to Asp)	**N331K** (Asn #331 to Lys)	**N331E** (Asn #331 to Glu)
**N333K** (Asn #333 to Lys)	**N333E** (Asn #333 to Glu)	**N349R** (Asn #349 to Arg)	**N349D** (Asn #349 to Asp)
**N351R** (Asn #351 to Arg)	**N351D** (Asn #351 to Asp)	**N349K** (Asn #349 to Lys)	**N349E** (Asn #349 to Glu)
**N351K** (Asn #351 to Lys)	**N351E** (Asn #351 to Glu)	**N365R** (Asn #365 to Arg)	**N365D** (Asn #365 to Asp)
**N339R** (Asn #339 to Arg)	**N339D** (Asn #339 to Asp)	**N365K** (Asn #365 to Lys)	**N365E** (Asn #365 to Glu)
**N339K** (Asn #339 to Lys)	**N339E** (Asn #339 to Glu)	**N319R** (Asn #319 to Arg)	**N319D** (Asn #319 to Asp)
**N331R** (Asn #331 to Arg)	**N331D** (Asn #331 to Asp)	**N319K** (Asn #319 to Lys)	**N319E** (Asn #319 to Glu)

**Table 4:** Aspartic Acid (Asp/D) amino acid mutants.

Enhancer	
**D335R** (Asp #335 to Arg)	**D335K** (Asp #335 to Lys)

**Table 5:** Cysteine (Cys/C) amino acid mutants.

Enhancer	Inhibitor
**C338R** (Cys #338 to Arg)	**C338D** (Cys #338 to Asp)
**C338K** (Cys #338 to Lys)	**C338E** (Cys #338 to Glu)
**C320R** (Cys #320 to Arg)	**C320D** (Cys #320 to Asp)
**C320K** (Cys #320 to Lys)	**C320E** (Cys #320 to Glu)
**C317R** (Cys #317 to Arg)	**C317D** (Cys #317 to Asp)
**C317K** (Cys #317 to Lys)	**C317E** (Cys #317 to Glu)
**C341R** (Cys #341 to Arg)	**C341D** (Cys #341 to Asp)
**C341K** (Cys #341 to Lys)	**C341E** (Cys #341 to Glu)

**Table 6:** Glutamine (Gln/Q) amino acid mutants.

Enhancer	
**Q321A** (Gln #321 to Ala)	**Q362A** (Gln #362 to Ala)
**Q321M** (Gln #321 to Met)	**Q362M** (Gln #362 to Met)
**Q321N** (Gln #321 to Asn)	**Q362N** (Gln #362 to Asn)
**Q321P** (Gln #321 to Pro )	**Q362P** (Gln #362 to Pro )
**Q321R** (Gln #321 to Arg)	**Q362R** (Gln #362 to Arg)
**Q321S** (Gln #321 to Ser )	**Q362S** (Gln #362 to Ser )
**Q321W** (Gln #321 to Trp )	**Q362W** (Gln #362 to Trp)
**Q321V** (Gln #321 to Val)	**Q362V** (Gln #362 to Val)
**Q321Y** (Gln #321 to Tyr )	**Q362Y** (Gln #362 to Tyr )
**Q321T** (Gln #321 to Thr)	**Q362T** (Gln #362 to Thr)

**Table 7:** Leucine (Leu/L) amino acid mutants.

Enhancer	Inhibitor
**L343D** (Leu #343 to Arg)	**L343D** (Leu #343 to Asp)
**L343K** (Leu #343 to Lys)	**L343E** (Leu #343 to Glu)
**L327R** (Leu #327 to Arg)	**L327D** (Leu #327 to Asp)
**L327K** (Leu #327 to Lys)	**L327E** (Leu #327 to Glu)
**L347R** (Leu #347 to Lys)	**L347D** (Leu #347 to Glu)
**L347K** (Leu #347 to Lys)	**L347E** (Leu #347 to Glu)
**L354R** (Leu #354 to Lys)	**L354D** (Leu #354 to Glu)
**L354K** (Leu #354 to Lys)	**L354E** (Leu #354 to Glu)

**Table 8:** Methionine (Met/M) amino acid mutants.

Enhancer	Inhibitor
**M356R** (Met #356 to Arg)	**M356D** (Met #356 to Asp)
**M356K** (Met #356 to Lys)	**M356E** (Met #356 to Glu)

**Table 9:** Threonine (Thr/T) amino acid mutants.

Enhancer	Inhibitor	Enhancer	Inhibitor
**T315R** (Thr #315 to Arg)	**T315D** (Thr #315 to Asp)	**T323R** (Thr #323 to Arg)	**T323D** (Thr #323 to Asp)
**T315K** (Thr #315 to Lys)	**T315E** (Thr #315 to Glu)	**T323K** (Thr #323 to Lys)	**T323E** (Thr #323 to Glu)
**T322R** (Thr #322 to Arg)	**T322D** (Thr #322 to Asp)	**T355R** (Thr #355 to Arg)	**T355D** (Thr #355 to Asp)
**T322K** (Thr #322 to Lys)	**T322E** (Thr #322 to Glu)	**T355K** (Thr #355 to Lys)	**T355E** (Thr #355 to Glu)
**T363R** (Thr #363 to Arg)	**T363D** (Thr #363 to Asp)	**T324R** (Thr #324 to Arg)	**T324D** (Thr #324 to Asp)
**T363K** (Thr #363 to Lys)	**T363E** (Thr #363 to Glu)	**T324K** (Thr #324 to Lys)	**T324E** (Thr #324 to Glu)
**T326R** (Thr #326 to Arg)	**T326D** (Thr #326 to Asp)	**T325R** (Thr #325 to Arg)	**T325D** (Thr #325 to Asp)
**T326K** (Thr #326 to Lys)	**T326E** (Thr #326 to Glu)	**T325K** (Thr #325 to Lys)	**T325E** (Thr #325 to Glu)

**Table 10:** *DNA* bases mutants.

Inhibitor	Inhibitor	Inhibitor	Inhibitor	Inhibitor
**G6T** (Gua #6 to Thy)	**T8A** (Thy #8 to Ade)	**T16A** (Thy #16 to Ade)	**A8T** (Ade #8 to Thy)	**A1T** (Ade #1 to Thy)
**G6A** (Gua #6 to Ade)	**T7G** (Thy #7 to Gua)	**T7C** (Thy #7 to Cyt)	**A6T** (Ade #6 to Thy)	**A1C** (Ade #1 to Cyt)
**G5T** (Gua #5 to Thy)	**A5G** (Ade #5 to Gua)	**G19C** (Gua #19 to Cyt)	**A7C** (Ade #7 to Cyt)	**A2G** (Ade #2 to Gua)
**G6C** (Gua #6 to Cyt)	**T16G** (Thy #16 to Gua)	**A9T** (Ade #9 to Thy)	**A8G** (Ade #8 to Gua)	**A2T** (Ade #2 to Thy)
**G5A** (Gua #5 to Ade)	**T8C** (Thy #8 to Cyt)	**A5C** (Ade #5 to Cyt)	**A16T** (Ade #16 to Thy)	**A9G** (Ade #9 to Gua)
**G5C** (Gua #5 to Cyt)	**A7T** (Ade #7 to Thy)	**A9G** (Ade #9 to Gua)	**C17T** (Cyt #17 to Thy)	**A11G** (Ade #11 to Gua)
**T18G** (Thy #18 to Gua)	**G19T** (Gua #19 to Thy)	**A15T** (Ade #15 to Thy)	**A6G** (Ade #8 to Gua)	**A11T** (Ade #11 to Thy)
**T8G** (Thy #8 to Gua)	**T17G** (Thy #17 to Gua)	**T16C** (Thy #16 to Cyt)	**A15G** (Ade #15 to Gua)	**C12T** (Cyt #12 to Thy)
**A5T** (Ade #5 to Thy)	**A9T** (Ade #9 to Thy)	**G19A** (Gua #19 to Ade)	**T17C** (Thy #17 to Cyt)	**C12G** (Cyt #12 to Gua)
**T18C** (Thy #18 to Cyt)	**A7G** (Ade #7 to Gua)	**T17A** (Thy #17 to Ade)	**A10T** (Ade #10 to Thy)	**C12A** (Cyt #12 to Ade)
**T18A** (Thy #18 to Ade)	**T7A** (Thy #7 to Ade)	**A9G** (Ade #9 to Gua)	**A1G** (Ade #1 to Gua)	**C20T** (Cyt #20 to Thy)
**C20G** (Cyt #20 to Gua)	**C20A** (Cyt #20 to Ade)			

As shown in Figs. 2, 3, 4, 5, 6, 7, 8, 9 and 10, the strength of each perturbation (mutation) is illustrated in terms of ΔΔG free energy calculation (Eq. 1a). Mutations of *acidic GATA3* residues or *DNA* bases have free energy values higher than the parent/wild free energy and are called *enhancers* because they enhance binding, whereas mutations of *basic GATA3* residues or *DNA* bases have free energy values lower than the parent/wild free energy and are called *inhibitors* because they inhibit binding. The lower the computed ΔΔG for a specific mutation, the more crucial the corresponding residue or *DNA* base is to binding, compared to other residues or *DNA* bases respectively, and the reasoning behind this is as follows: If the computed *solvation* binding free energy ΔΔG of a mutated residue or *DNA* base is lower than the initial *solvation* binding free energy ΔΔG of the same residue or *DNA* base before mutation, this implies that this specific residue/*DNA* base binds better before being mutated, and so this residue/*DNA* base has a significant impact on binding and is considered a hotspot (case of an *inhibitor*). Conversely, if the computed *solvation* binding free energy ΔΔG of a mutated residue or *DNA* base is higher than the initial *solvation* binding free energy ΔΔG of the same residue or *DNA* base before mutation, this implies that this specific residue/*DNA* base binds better after being mutated, and so this residue/*DNA* base does not have a significant impact on binding (case of an *enhancer*).

In Fig. 10, all mutants of all bases of the double-stranded Subsequence_1__*GATA*_Subsequence_2_ *DNA* sequence are studied. Since each strand (-Y or -Z) of the *DNA* sequence is numbered from 1 to 20 (in the .pdb file), we are focusing on the impact of each base of the *DNA* sequence where *GATA3* protein actually binds. Accordingly, we are looking at bases located at positions 4–5–6–7–8–9 on Strand-Y where Chain-C binds, corresponding to nucleotides T**GATA**A, and to complementary subsequence TTATCA on Strand-Z at positions 14–15–16–17–18–19. On the other hand, we are looking at bases located at positions 5–6–7–8–9–10 on Strand-Z where Chain-D binds, corresponding to nucleotides AGATAA, and to complementary subsequence of TTATCT on Strand-Y at positions 13–14–15–16–17–18 (as shown below with underlines and blue highlights).

**DNA Strand-Z:** A_1_A_2_G_3_C_4_A_5_**G**_**6**_**A**_**7**_**T**_**8**_**A**_**9**_A_10_G_11_T_12_C_13_T_14_T_15_A_16_T_17_C_18_A_19_G_20_.

**DNA Strand-Y:** C_20_G_19_T_18_C_17_T_16_A_15_T_14_T_13_C_12_A_11_G_10_A_9_**A**_**8**_**T**_**7**_**A**_**6**_**G**_**5**_T_4_C_3_T_2_T_1_.

The results showed the following *DNA* bases mutants to impact binding (inhibitors shown below with green highlights). The lowest free energy values correspond to inhibitors with highest impact (strongest inhibitors), and those are: **G6T**, **G6A**, and **G6C** on Strand-Z, and **G5T**, **G5A**, and **G5C** on Strand-Y. Accordingly, we can see how the bases in the ‘*GATA*’ subsequence are definitely the first to impact binding, and then a few bases in the neighborhood of the ‘*GATA*’ subsequence, which are not necessarily the first adjacent base on the left and/or the first adjacent base on the right of the ‘*GATA*’ subsequence. The rest of the DNA bases mutants that lay around the parent/wild region (Fig. 10) do not have a major impact on binding.

**DNA Strand-Z:** A_1_A_2_G_3_C_4_A_5_**G**_**6**_**A**_**7**_**T**_**8**_**A**_**9**_A_10_G_11_T_12_C_13_T_14_T_15_A_16_T_17_C_18_A_19_G_20_ (Chain-D – Chain-C).

**DNA Strand-Y:** C_20_G_19_T_18_C_17_T_16_A_15_T_14_T_13_C_12_A_11_G_10_A_9_**A**_**8**_**T**_**7**_**A**_**6**_**G**_**5**_T_4_C_3_T_2_T_1_ (Chain-D – Chain-C).

**Figure 11:**
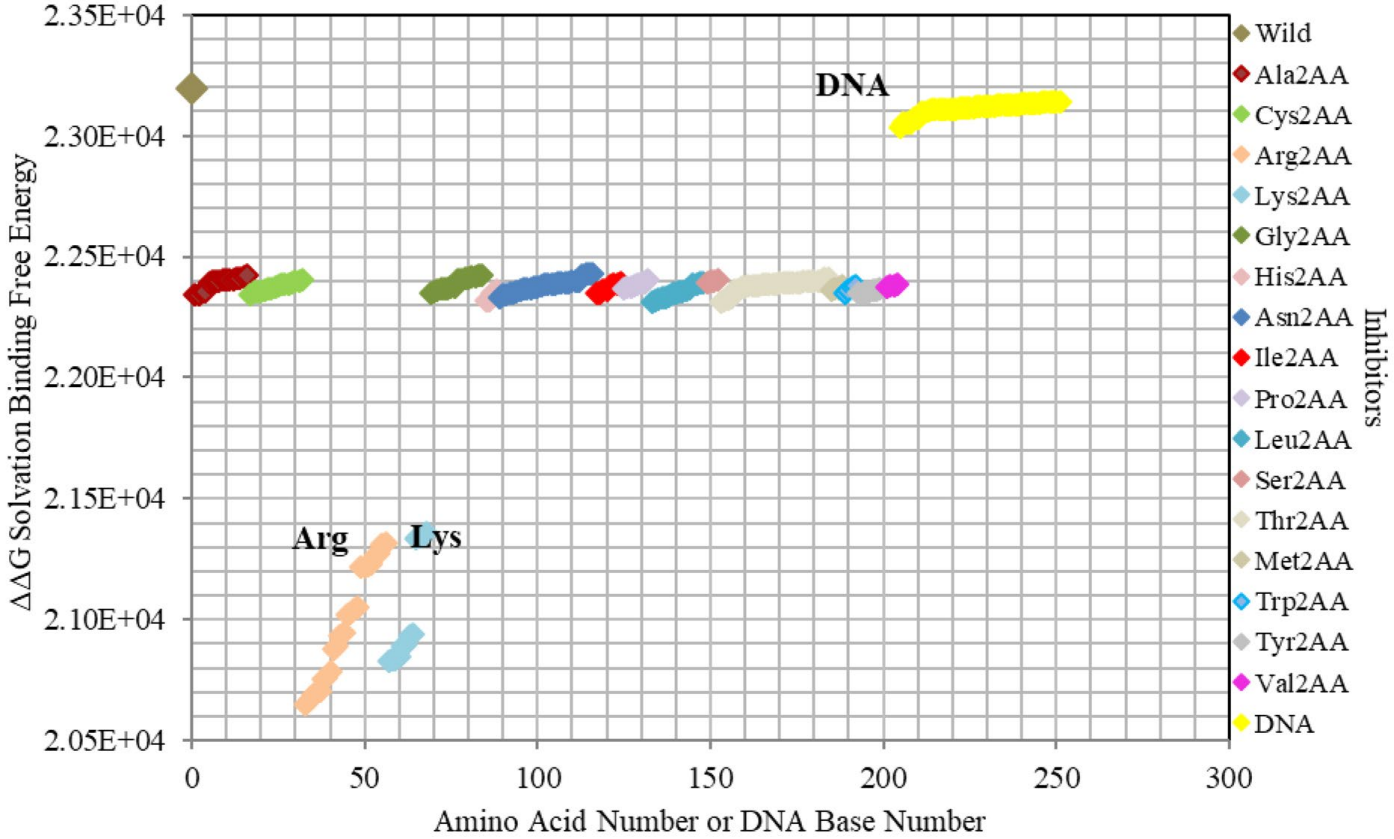
Electrostatic free energy differences of all *GATA3 inhibitors* within *GATA3:DNA* complex. Plot presents the solvated binding free energy calculations (in KJ/mol) of all *GATA3* amino acid mutants that are inhibitors in both of Chain-C and Chain-D, and that is in comparison with those of the *DNA* sequence bases in order to elucidate their relative effect on binding.

**Figure 12:**
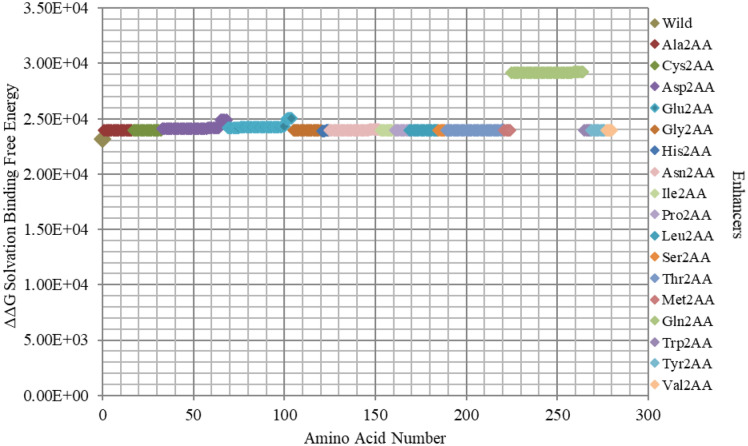
Electrostatic free energy differences of all *GATA3 enhancers* within *GATA3:DNA* complex. Plot presents the solvated binding free energy calculations (in KJ/mol) of all *GATA3* amino acid mutants that are enhancers in both of Chain-C and Chain-D.

Comparatively, all *inhibitors* are presented together in order to reveal the ones with the highest impact on protein-*DNA* binding. Hence, Fig. 11 shows that Arg and Lys are the most influential amino acids for efficient binding. It also shows the minor impact of mutated *DNA* bases, which lay around the parent/wild region, when compared to amino acid mutants. This result uncovers the crucial role of *DNA* backbone in the interactions with the *GATA3* protein amino acids, unlike the specific role of the *DNA* bases, which appears to be minimal in comparison. Similarly, Fig. 12 shows all enhancers of amino acids, highlighting the crucial role of Gln mutants in binding.

Computationally, we detected the hydrogen bonds (listed in detail in in Supplementary Figure SF12 and Supplementary Table ST12) between Chain-C and the *DNA*, revealing the vital role to binding of the following amino acids: Arg312, Arg329, Arg330, Arg364, Arg366, Lys346, and Asn339. In addition, we computationally detected the salt bridges (listed in detail in in Supplementary Figure SF13 and Supplementary Table ST13) between Chain-C and the *DNA* for the following amino acids: Arg312, Arg330, Arg352, and Lys358.

Mutating amino acids to Arg or Lys make them enhancers due to adding more positive to the binding with *DNA*, which is initially more negatively charged. Figures 2, 3, 4, 5, 6, 7, 8 and 9 show the effect on binding when mutating any amino acid to Arg or Lys. Conversely, when any amino acid is mutated to Asp/Glu, it is implied as an inhibitor that impedes binding. In particular, when Arg or Lys are changed to Asp/Glu (Fig. 2/Table 2 or SF6/ST6 respectively), those mutants are called strong inhibitors and the impeding effect on binding is multiplied. In this case, we are losing more positive charges (property of Arg and Lys) and adding more negative charges (property of Asp and Glu), and that is not favorable for binding to *DNA* which has the property of being negatively charged on the backbone.

### Experimental validation

Our computational results are further validated against experimental evidence, based on experimental published papers^16^. The experimental information, listed in Supplementary Tables ST14, ST15, ST16, and ST17, substantiates our mutational results in Figs. 2, 3, 4, 5, 6, 7, 8, 9, 10, 11 and 12 and in SF1-SF11. The crucial effect of the predicted key amino acids and key *DNA* bases on binding are verified through: (1) The different *GATA3* crystal structure models available, (2) the hub of protein–protein and/or protein-*DNA* interactions they are engaged in, and (3) The association in diseases and manifestation in phenotypes.

#### ADJacent and OPPosite GATA3:DNA models

The complex *GATA3:DNA* exists in two forms: The Opposite model (OPP), as shown in Fig. 1a, and the Adjacent model (ADJ), as shown in Fig. 1b. The OPP model (PDB: 3DFX) has *GATA3* factors Chain-A and Chain-B binding to opposite ends of the double stranded *DNA* (Strand-X and -Y) and the ADJ model (PDB: 3DFX) has *GATA3* factors Chain-C and Chain-D binding to same ends of the double stranded *DNA* (Strand-Y and -Z).

In^43^, the actual binding of *GATA3* to *DNA* lies in the second chain from the N-terminal, which is Chain-B in the OPP model and Chain-C in the ADJ model. In both models, Chain-A (OPP model) and Chain-D (ADJ model) bind to Friends of *GATA* (FOG); their role is only to enhance binding of *GATA3* to DNA.

Both models (OPP and ADJ) are similar in that Chain-B and Chain-C both bind to ‘*GATA*’ *DNA* subsequence. The only minor difference between Chain-A and Chain-D lies in that they bind to slightly different *DNA* subsequences, ‘*GATT*’ and ‘*GATA*’ respectively. Hence, we can use some of the experimental results performed on the OPP model to validate some of the computational results performed on the ADJ model. As shown in Fig. 1a, Chain-A binds to ‘*GATT*’ bases at positions 13, 14, 15, and 16 of the *DNA* Strand-Y, and Chain-B binds to ‘*GATA*’ bases at positions 14, 15, 16, and 17 of the *DNA* Strand-X. Figure 1b shows Chain-D binding to ‘*GATA*’ bases at positions 6, 7, 8, and 9 of the *DNA* Strand-Z and Chain-C binding to ‘*GATA*’ bases at positions 5, 6, 7, and 8 of the *DNA* Strand-Y.

#### Interactions and hubs

Based on the results of our *Expanded*-AESOP method (as shown in Figs. 2, 3, 4, 5, 6, 7, 8, 9 and 10), we validated experimentally the predicted enhancers and inhibitors, described them in details in Supplementary Material, and summarized them in Supplementary Tables ST14, ST15, ST16, and ST17. Experimental evidence showed extensive protein–protein interactions (between the two *GATA3* molecules) and protein-*DNA* interactions (between *GATA3* and the *DNA* molecule) of many enhancers (**Ala340, Asn351, Asn349, Asn339, Pro353, Leu354, Leu347, Leu327**, **Leu343, Glu359, His348, Ile350, Ile361, Thr355, Thr326, Gln362, Met356**, **Tyr344)** and many inhibitors (**Ala340, Arg364, Arg329, Arg352, Arg312, Arg330, Lys357, Lys358, Lys346, Asn351, Asn349, Asn339, Pro353, Leu354, Leu347, Leu327, Leu343, His348, Ile350, Ile361, Thr355, Thr326, Met356**, **Tyr344**), revealing strong hubs of interactions comprising all different types of interactions (hydrogen bonds, salt bridges, van der Waals, etc.).

On the other hand, we detected computationally hydrogen bonds between Chain-C amino acids (**Arg312, Arg329, Arg330, Arg364, Arg366, Lys346, Asn339)** and the DNA (shown in Supplementary Figure SF12 and summarized in Supplementary Table ST12). In addition, we detected computationally salt bridges between Chain-C amino acids (**Arg312, Arg330**, **Arg352, Lys358)** and the DNA (shown in SF13 and ST13), again bringing to light the role of those amino acids to binding.

Thus, the experimental and computational validations substantiate the crucial role of the predicted amino acids and of the *DNA* backbone, in addition to demonstrating the effectiveness of our developed approach *Expanded*-AESOP.

#### Gene-disease associations/phenotypes

Identifying gene-disease associations from experimental methods can be expensive and time consuming. Yet, this process is highly needed to design therapeutic strategies against diseases. Accordingly, in silico methods were developed to predict those associations from available experimental data and other types of data. In this section, we validated the results predicted by our computational method by elaborating on the diseases already witnessed as a result of the disruptions caused by our studied mutations.

The enhancer **C317R** (as listed in the *GATA3:DNA* .pdb file from the Protein Data Bank, but numbered as **C318R** in^12^—with an offset of one position) caused by a missense mutation as shown in Fig. 5, leads to a disruption of the second zinc finger (*ZnF2*) that is manifested in HDR syndrome, where the loss of *ZnF2* coordination was marked as haploinsufficiency (HI)^12^. Another missense mutation, leading to a disruption of the second zinc finger (ZnF2) and manifesting in HDR syndrome, is the enhancer **N319K** (numbered as **N320K** in^12^), as shown in Fig. 3. Again, this specific missense mutation has been noticeable as HI in the HDR syndrome^12^. A different missense mutation caused by the enhancer **L347R** (numbered as **L348R** in^11^), is shown in Fig. 7, and has been observed in the HDR syndrome^11^. This mutation affects the basic region and is likely to disturb the *DNA* conformational change. The mutation **R352S**^4^, despite being in the parent/wild region, has a major effect. As shown in Fig. 2, it is on the far end of the parent/wild region, and that makes it an approximate inhibitor. It is predicted to disrupt the helical turn and thus change the angle between the C-terminal zinc finger and the adjacent C-terminal tail; this phenomenon has been visible in the HDR syndrome within the Chinese population^15^.

On the other hand, *GATA3* TF is one of the most frequently mutated genes in Breast Cancer. The mutation **R366L** (numbered as **R367L** in^49^) which lies in the parent/wild region, turns to be of major effect. As shown in Fig. 2, it is considered an approximate inhibitor due to its distant location from the parent/wild complex. Such missense mutation in Exon5 of *ZnF2* was seen in Breast Tumor Ull-011^49^, resulting in high expression of *GATA3*, and leading to Breast Neoplasm disease^49^. Also, the missense mutation **L343F** (numbered as **L344F** in^49^) in Exon 5 of *ZnF2* showed high expression of *GATA3* in Breast Tumor BR00-0587, causing the same disease^49^. On the other hand, the mutations **M356K** (numbered as **M294K** in^50^ and is the only Methionine in *ZnF2*) and **N333K** (numbered as **N334K** in^50^), in Exon 4 and Exon 5 respectively, were witnessed in Breast Cancer on the molecular and clinical levels^50^. A heterozygous mutation (frameshift) **D335Gfs** (numbered as **D336Gfs** in^51^) was seen in Breast Cancer^51^ and the frameshift mutation **R329fs** (numbered as **R330fs** in^52,53^) was associated with Breast Cancer. The previous two results show the importance of the specified amino acids (D335 and R329) which were predicted as strong enhancer and inhibitor respectively in our results (Figs. 2 and 4 respectively). Lastly, the mutational frameshift **P490Afs**, which occurred in several Breast Cancer cases^54^, shows the importance of studying all *GATA3* mutants (charged and non-charged amino acids). Yet, we could not verify this specific amino acid (P490) due to its position outside *ZnF2* (not covered in .pdb input file range).

## Conclusion

We started from the AESOP^26,27,29,33–42^ framework, which predicts *ionic* residues with major effect to binding, and modified it to the *Expanded*-AESOP framework, which predicts all types of residues (*ionic and non-ionic*) and *DNA* bases, affecting binding in a biomolecular protein-*DNA* complex. Unlike previous *GATA* work^51–55^ where we tackled only *charged* amino acids, we modified the method here to cover all mutations types of all amino acids and all mutations types of all *DNA* bases. We applied the new method to the structural information of two models of the *GATA3:DNA* complex^43^. After computing the electrostatic potential calculations using APBS, and feeding them into the free energy calculations in view of a two-step model, we detected key residues and key *DNA* bases crucial for the complex intermolecular interactions, and therefore for binding. Analysis of the corresponding free energy calculations showed that the *DNA* backbone plays a more critical role in binding than the *DNA* bases, and that was confirmed by the related interactions listed computationally and experimentally.

The results showed that some *non-ionic* amino acids do play a major role in binding, and that may be rationalized to many factors, such as the position of the *non-ionic* amino acid in the complex (i.e., too close to the interface or too close to many other charged amino acids) or the contribution of this *non-ionic* amino acid to some favorable conformation.

Future work will include studying key amino acids and key *DNA* bases in the crystal structure of *GATA4:DNA*. Such studies will form the basis for designing future experiments and biopharmaceutical studies that will assist in understanding better the biochemical pathways involved in *GATA:DNA* binding, for enhanced regulation of *GATA* target genes.

## Supplementary Information

Supplementary Information.
