# Supplementary material for: Mutational signatures in GATA3 transcription factor and its DNA binding domain that stimulate breast cancer and HDR syndrome
Source: Sci Rep. 2021 Nov 23;11:22762. doi: 10.1038/s41598-021-01832-z (PMC8611019; doi:10.1038/s41598-021-01832-z)
Supplement: Supplementary file 1 — Supplementary Information. [file 41598_2021_1832_MOESM1_ESM.docx]

**Mutational Signatures in GATA3 Transcription Factor and its DNA Binding Domain that Stimulate Breast Cancer and HDR Syndrome**

**Nature Scientific Reports**

Atlal El-Assaad^1ac*^, Zaher Dawy^1a^, Athar Khalil^2b^, and Georges Nemer^2b^

^a^Department of Electrical and Computer Engineering, American University of Beirut (AUB), Riad El Solh, Beirut, Lebanon

^b^Department of Biochemistry and Molecular Genetics, American University of Beirut (AUB), Riad El Solh, Beirut, Lebanon

^c^Department of Computer Science, Lebanese International University (LIU), Bekaa, Lebanon

atlal.assaad@liu.edu.lb, [zd03@aub.edu.lb](mailto:zd03@aub.edu.lb), [aak67@mail.aub.edu](mailto:aak67@mail.aub.edu), gn08@aub.edu.lb

***Address correspondence to:**

Atlal M. El-Assaad

Department of Computer Science, Lebanese International University, Bekaa - Lebanon.

Electronic mail: [atlal.assaad@liu.edu.lb](about:blank)

Telephone Number: 961-76-163245

ORCID: 0000-0003-2872-699X


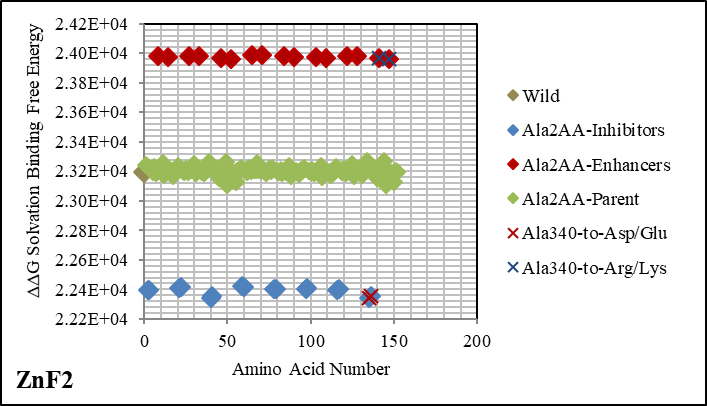


**Supplementary Figure 1 (SF1). Electrostatic free energy differences of Alanine (Ala/A)** **within *GATA3:DNA* complex.** Plot presents the solvated binding free energy calculations (in KJ/mol) of *GATA3* Ala amino acid mutants in both of Chain-D and Chain-C.

**Supplementary Table 1 (ST1). Alanine (Ala/A) amino acid mutants**

| **Enhancer** | **Inhibitor** |
| --- | --- |
| **A340R** (Ala #340 to Arg) | **A340D** (Ala #340 to Asp) |
| **A340K** (Ala #340 to Lys) | **A340E** (Ala #340 to Glu) |
| **A332R** (Ala #332 to Arg) | **A332** (Ala #332 to Asp) |
| **A332K** (Ala #332 to Lys) | **A332E** (Ala #332 to Glu) |
| **A318R** (Ala #318 to Arg) | **A318D** (Ala #318 to Asp) |
| **A318K** (Ala #318 to Lys) | **A318E**(Ala #318 to Glu) |
| **A313R** (Ala #313 to Arg) | **A313D** (Ala #313 to Asp) |
| **A313K** (Ala #313 to Lys) | **A313E** (Ala #313 to Glu) |


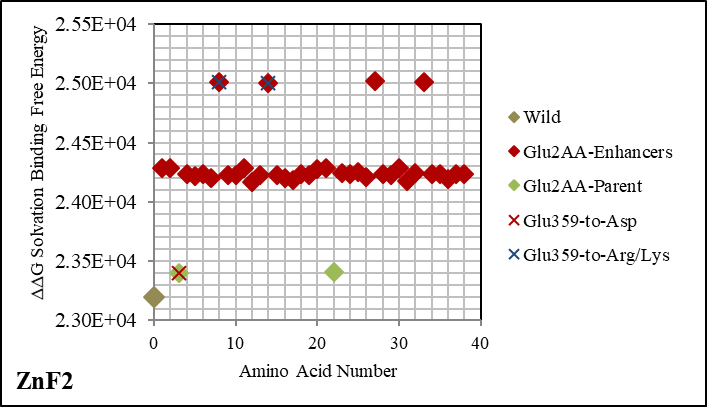


**Supplementary Figure 2 (SF2). Electrostatic free energy differences of Glutamic Acid (Glu/E)** **within *GATA3:DNA* complex.** Plot presents the solvated binding free energy calculations (in KJ/mol) of *GATA3* Glu amino acid mutants in both of Chain-D and Chain-C.

**Supplementary Table 2 (ST2). Glutamic Acid (Glu/E) amino acid mutants**

| **Enhancer** | |
| --- | --- |
| **E359R** (Glu #359 to Arg) | **E359K** (Glu #359 to Lys) |


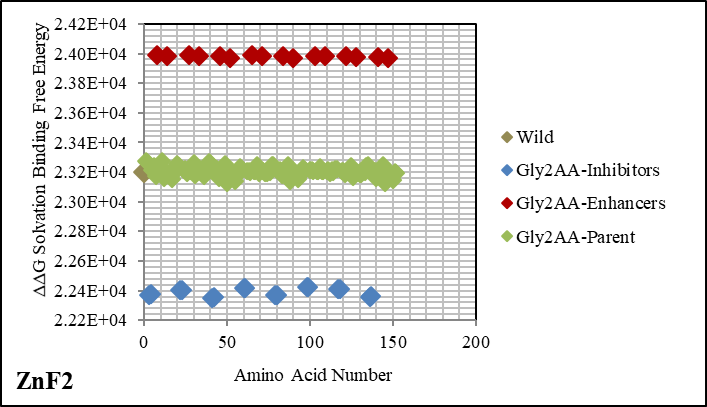


**Supplementary Figure 3 (SF3). Electrostatic free energy differences of Glycine (Gly/G)** **within *GATA3:DNA* complex.** Plot presents the solvated binding free energy calculations (in KJ/mol) of *GATA3* Gly amino acid mutants in both of Chain-D and Chain-C.

**Supplementary Table 3 (ST3). Glycine (Gly/G) amino acid mutants**

| **Enhancer** | **Inhibitor** |
| --- | --- |
| **G342R** (Gly #342 to Arg) | **G342D** (Gly #342 to Asp) |
| **G342K** (Gly #342 to Lys) | **G342E** (Gly #342 to Glu) |
| **G360R** (Gly #360 to Arg) | **G360D** (Gly #360 to Asp) |
| **G360K** (Gly #360 to Lys) | **G360E** (Gly #360 to Glu) |
| **G334R** (Gly #334 to Arg) | **G334D** (Gly #334 to Asp) |
| **G334K** (Gly #334 to Lys) | **G334E** (Gly #334 to Glu) |
| **G314R** (Gly #314 to Arg) | **G314D** (Gly #314 to Asp) |
| **G314K** (Gly #314 to Lys) | **G314E** (Gly #314 to Glu) |


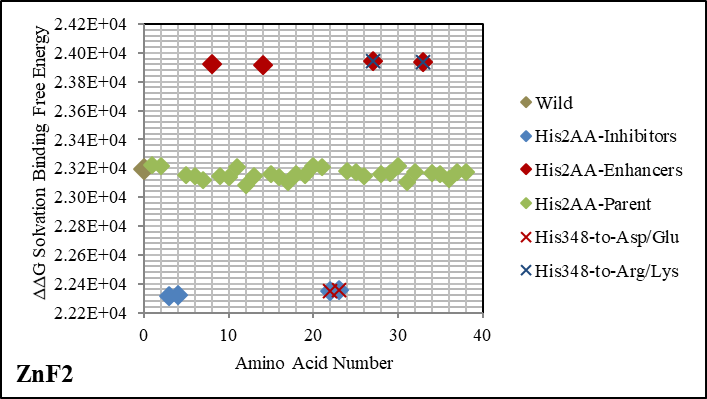


**Supplementary Figure 4 (SF4). Electrostatic free energy differences of Histidine (His/H)** **within *GATA3:DNA* complex.** Plot presents the solvated binding free energy calculations (in KJ/mol) of *GATA3* His amino acid mutants in both of Chain-D and Chain-C.

**Supplementary Table 4 (ST4). Histidine (His/H) amino acid mutants**

| **Enhancer** | **Inhibitor** |
| --- | --- |
| **H348R** (His #348 to Arg) | **H348D** (His #348 to Asp) |
| **H348K** (His #348 to Lys) | **H348E** (His #348 to Glu) |


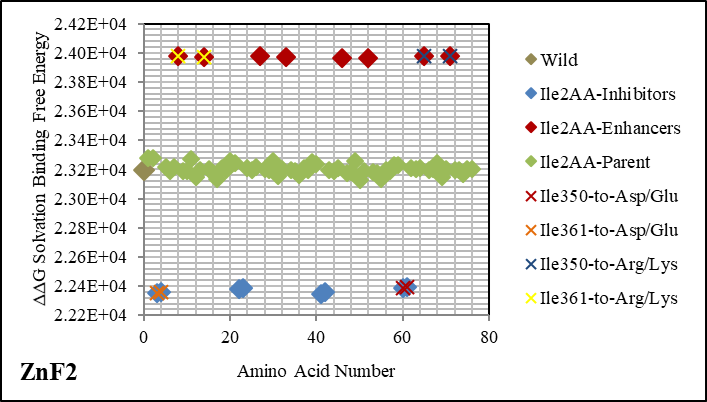


**Supplementary Figure 5 (SF5). Electrostatic free energy differences of Isoleucine (Ile/I)** **within *GATA3:DNA* complex.** Plot presents the solvated binding free energy calculations (in KJ/mol) of *GATA3* Ile amino acid mutants in both of Chain-D and Chain-C.

**Supplementary Table 5 (ST5). Isoleucine (Ile/I) amino acid mutants**

| **Enhancer** | **Inhibitor** |
| --- | --- |
| **I361R** (Ile #361 to Arg) | **I361D** (Ile #361 to Asp) |
| **I361K** (Ile #361 to Lys) | **I361E** (Ile #361 to Glu) |
| **I350R** (Ile #350 to Arg) | **I350D** (Ile #350 to Asp) |
| **I350K** (Ile #350 to Lys) | **I350E** (Ile #350 to Glu) |


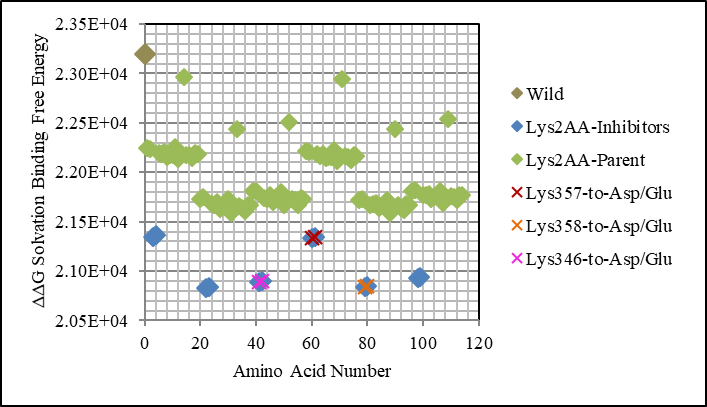


**Supplementary Figure 6 (SF6). Electrostatic free energy differences of Lysine (Lys/K)** **within *GATA3:DNA* complex.** Plot presents the solvated binding free energy calculations (in KJ/mol) of *GATA3* Lys amino acid mutants in both of Chain-D and Chain-C.

**Supplementary Table 6 (ST6). Lysine (Lys/K) amino acid mutants**

| **Inhibitor** | |
| --- | --- |
| **K358D** (Lys #358 to Asp) | **K346E** (Lys #346 to Glu) |
| **K358E** (Lys #358 to Glu) | **K357D** (Lys #357 to Asp) |
| **K346D** (Lys #346 to Asp) | **K357E** (Lys #357 to Glu) |


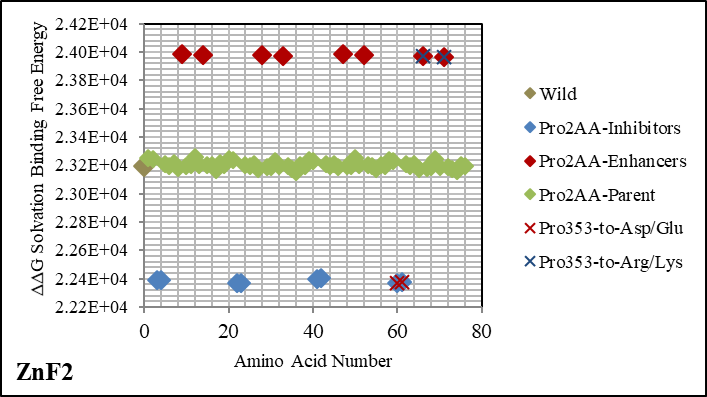


**Supplementary Figure 7 (SF7). Electrostatic free energy differences of Proline (Pro/P)** **within *GATA3:DNA* complex.** Plot presents the solvated binding free energy calculations (in KJ/mol) of *GATA3* Pro amino acid mutants in both of Chain-D and Chain-C.

**Supplementary Table 7 (ST7). Proline (Pro/P) amino acid mutants**

| **Enhancer** | **Inhibitor** |
| --- | --- |
| **P353R** (Pro #353 to Arg) | **P353D** ( Pro #353 to Asp) |
| **P353K** (Pro #353 to Lys) | **P353E** (Pro #353 to Glu) |
| **P336R** (Pro #336 to Arg) | **P336D** (Pro #336 to Asp) |
| **P336K** (Pro #336 to Lys) | **P336E** (Pro #336 to Glu) |


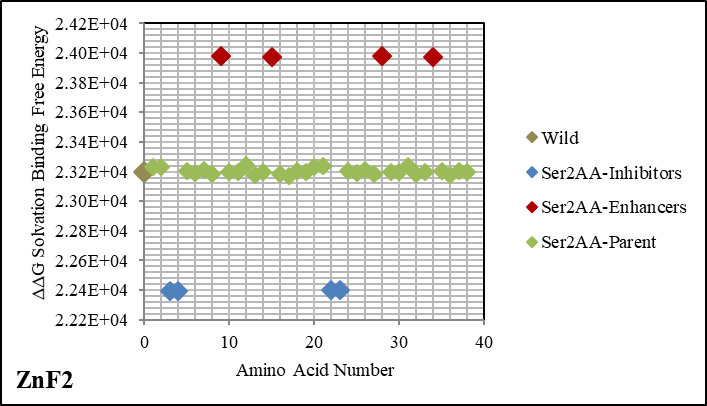


**Supplementary Figure 8. Electrostatic free energy differences of Serine (Ser/S)** **within *GATA3:DNA* complex.** Plot presents the solvated binding free energy calculations (in KJ/mol) of *GATA3* Ser amino acid mutants in both of Chain-D and Chain-C.

**Supplementary Table 8 (ST8). Serine (Ser/S) amino acid mutants**

| **Enhancer** | **Inhibitor** |
| --- | --- |
| **S316R** (Ser #316 to Arg) | **S316D** ( Ser #316 to Asp) |
| **S316K** (Ser #316 to Lys) | **S316E** (Ser #316 to Glu) |


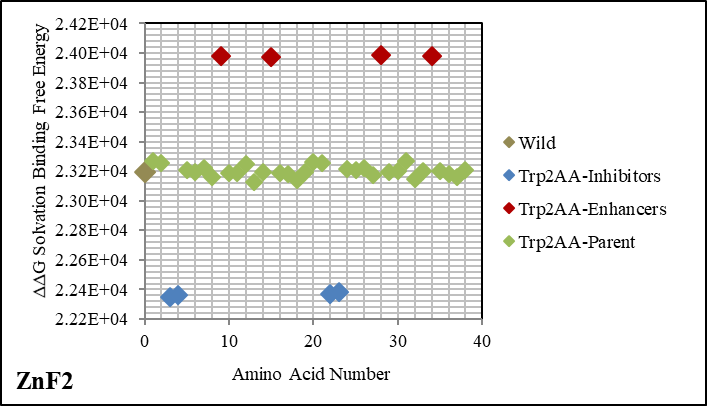


**Supplementary Figure 9 (SF9). Electrostatic free energy differences of Tryptophan (Trp/W)** **within *GATA3:DNA* complex.** Plot presents the solvated binding free energy calculations (in KJ/mol) of *GATA3* Trp amino acid mutants in both of Chain-D and Chain-C.

**Supplementary Table 9 (ST9). Tryptophan (Trp/W) amino acid mutants**

| **Enhancer** | **Inhibitor** |
| --- | --- |
| **W328R** (Trp #328 to Arg) | **W328D** (Trp #328 to Asp) |
| **W328K** (Trp #328 to Lys) | **W328E** (Trp #328 to Glu) |


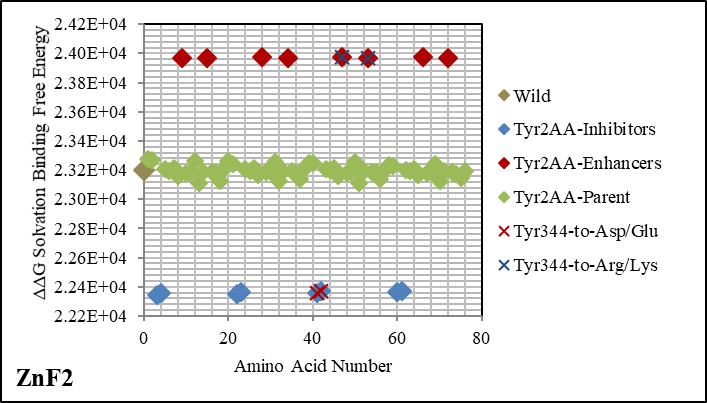


**Supplementary Figure 10 (SF10). Electrostatic free energy differences of Tyrosine (Tyr/W)** **within *GATA3:DNA* complex.** Plot presents the solvated binding free energy calculations (in KJ/mol) of *GATA3* Tyr amino acid mutants in both of Chain-D and Chain-C.

**Supplementary Table 10 (ST10). Tyrosine (Tyr/Y) amino acid mutants**

| **Enhancer** | **Inhibitor** |
| --- | --- |
| **Y344R** (Tyr #344 to Arg) | **Y344D** (Tyr #344 to Asp) |
| **Y344K** (Tyr #344 to Lys) | **Y344E** (Tyr #344 to Glu) |
| **Y345R** (Tyr #344 to Arg) | **Y345D** (Tyr #345 to Asp) |
| **Y345K** (Tyr #344 to Lys) | **Y345E** (Tyr #345 to Glu) |


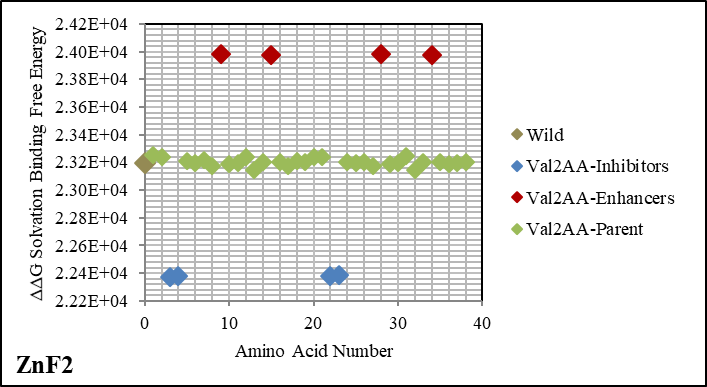


**Supplementary Figure 11 (SF11). Electrostatic free energy differences of Valine (Val/V)** **within *GATA3:DNA* complex.** Plot presents the solvated binding free energy calculations (in KJ/mol) of *GATA3* Val amino acid mutants in both of Chain-D and Chain-C.

**Supplementary Table 11 (ST11). Valine (Val/V) amino acid mutants**

| **Enhancer** | **Inhibitor** |
| --- | --- |
| **V337R** (Val #337 to Arg) | **V337D** (Val #337 to Asp) |
| **V337K** (Val #337 to Lys) | **V337E** (Val #337 to Glu) |

Computationally, we detected the following hydrogen bonds between Chain-C amino acids and the DNA (shown below in Supplementary Figure SF12 and summarized in Supplementary Table ST12), revealing the crucial role of amino acids to binding: **Arg312** at atom NH1 from Chain-C makes a hydrogen bond with C3 at atom OP2 from DNA Strand-Y, via a distance of 2.966Å. **Arg329** at atom NH2 from Chain-C makes a hydrogen bond with T17 at atom O4 from DNA Strand-Z, via a distance of 3.209Å. **Arg330** at atom NH2 from Chain-C makes a hydrogen bond with C3 at atom OP1 from DNA Strand-Y, via a distance of 2.87 Å. **Arg364** at atom NH1 from Chain-C makes a hydrogen bond with T15 at atom O2 from DNA Strand-Z, via a distance of 3.06 Å. **Arg366** at atom NH2 from Chain-C makes a hydrogen bond with T17 at atom O2 from DNA Strand-Z, via a distance of 2.927 Å. **Lys346** at atom NZ from Chain-C makes a hydrogen bond with G5 at atom OP2 from DNA Strand-Y, via a distance of 2.738 Å. **Asn339** at atom ND2 from Chain-C makes a hydrogen bond with A16 at atom N7 from DNA Strand-Z, via a distance of 3.256 Å. All the previously elaborated hydrogen bonds interactions highlight the crucial role of the positively charged amino acids Arg and Lys in binding to the negatively charged backbone of the DNA.

In addition, we detected computationally the following salt bridges between Chain-C amino acids and the DNA (shown in SF13 and ST13), revealing the crucial role of the following amino acids in binding: **Arg312** at atom CZ from Chain-C makes a salt bridge with C3 at atom OP1 from DNA Strand-Y, via a distance of 4.964 Å. **Arg330** at atom CZ from Chain-C makes a salt bridge with C3 at atom OP1 from DNA Strand-Y, via a distance of 3.128 Å. **Arg352** at atom CZ from Chain-C makes a salt bridge with T15 at atom OP1 from DNA Strand-Z, via a distance of 3.944 Å. **Lys358** at atom NZ from Chain-C makes a salt bridge with T15 at atom OP1 from DNA Strand-Z, via a distance of 4.84 Å. In addition, Lys358 at atom NZ from Chain-C makes a salt bridge with C12 at atom OP1 from DNA Strand-Y, with a distance of 4.184 Å. Once more, the previously mentioned salt bridge interactions show how Arg and Lys play crucial roles in the binding between GATA3 protein and DNA, especially since they are positively charged amino acids and DNA is negatively charged on the backbone.


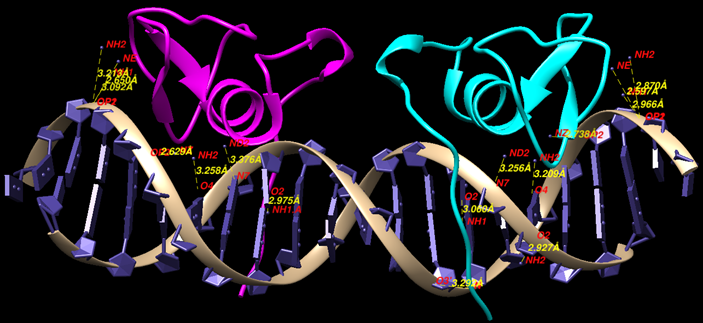


**Supplementary Figure 12 (SF12). Hydrogen bonds.** All Hydrogen bonds between Chain-D (magenta) and the double stranded (ds) *DNA*, and between Chain-C (cyan) and the *dsDNA*. Distances are labeled in Angstroms, where only interactions between Chain-C and the DNA are detailed in Table ST12.

**Supplementary Table 12 (ST12). Hydrogen Bonds from Computation (Illustration of SF12).** Hydrogen bonds are between amino acids in Chain-C and the double stranded DNA bases. Distances are measured in Angstroms.

|  | **C13** | **T17** | **C3** | **T15** | **T17** | **G5** | **A16** |
| --- | --- | --- | --- | --- | --- | --- | --- |
| **R312** | 2.966 |  |  |  |  |  |  |
| **R329** |  | 3.209 |  |  |  |  |  |
| **R330** |  |  | 2.87 |  |  |  |  |
| **R364** |  |  |  | 3.06 |  |  |  |
| **R366** |  |  |  |  | 2.927 |  |  |
| **K346** |  |  |  |  |  | 2.738 |  |
| **N339** |  |  |  |  |  |  | 3.256 |


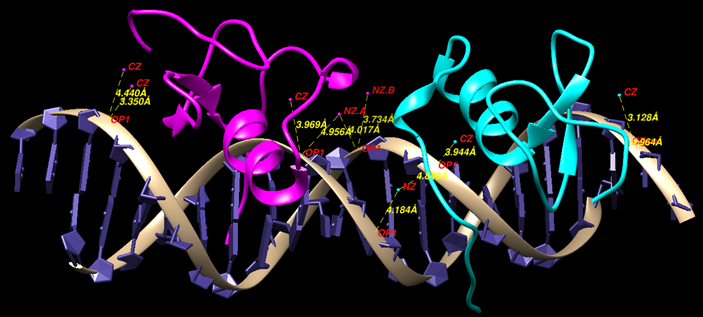


**Supplementary Figure 13 (SF13). Salt bridges.** All Salt Bridges bonds between Chain-D (magenta) and the double stranded (ds) DNA, and between Chain-C (cyan) and the dsDNA. Distances are labeled in Angstroms (less than 5Å) and detailed in Table ST13.

**Supplementary Table 13 (ST13). Salt Bridges from Computation (Illustration of SF13).** Salt bridges between amino acids in Chain-C and the double stranded DNA bases. Distances are less than 5 and measured in Angstroms.

|  | **C3** | **T15** | **C12** |
| --- | --- | --- | --- |
| **R312** | 4.964 |  |  |
| **R330** | 3.128 |  |  |
| **R352** |  | 3.944 |  |
| **K358** |  | 4.84 | 4.184 |

We validated experimentally the results of predicted enhancers and inhibitors, based on our *Expanded*-AESOP method (shown in Article Figs. 2-10), and summarized them in Supplementary Tables ST14, ST15, ST16, and ST17. For instance, **Ala340**, which is an enhancer and inhibitor in SF1, interacts with the sugar phosphate backbone of the DNA extensively, in addition to making contacts with the DNA bases (ST14). **Arg364**, which is an inhibitor in Fig. 2, makes base specific hydrogen bonds with the DNA, where the C-terminal basic tail inserts into the minor groove; it forms a hydrogen bond with the carbonyl of T6′. In addition, Arg364 makes extensive van der Waals contacts with neighboring bases and sugar rings (ST14). **Arg329**, which is an inhibitor in Fig. 2, makes direct hydrogen bonds with the DNA bases. On the consensus site of the OPP complex, Arg329 interacts with G14 through bidentate hydrogen bonds. It forms a hydrogen bond with T8’, and makes direct van der Waals contacts with DNA bases (ST14). **Arg352**, which is an inhibitor in Fig. 2, is one of the major amino acids that mediates protein-protein interactions in the C-terminal basic tail, whose conformational change is mostly determined by it and by other amino acids that might interact with it, like Tyr344, Tyr345, His348, and Met356. In addition, Arg352 interacts with the DNA backbone and minor groove extensively, and as a result, forms an extended protein-DNA and protein–protein interaction interface. Arg352 binds to alpha helix through numerous hydrogen bonds & vdW contacts (ST14, ST15, ST17).

**Arg312 and Arg330**, which are inhibitors in Fig. 2, interact with the sugar phosphate backbone of the DNA extensively, in addition to making contacts with DNA bases (ST14). **Lys357 and Lys358** of the second half of the C-terminal basic tail of one finger, which are inhibitors in SF6, interact with residues Leu347, His348, Asn349, and Ile350 at the end of the recognition helix of the other finger, causing minor conformation to the C-terminal. In addition, they interact with the DNA backbone and minor groove extensively, hence forming an extended protein-DNA and protein–protein interaction interface (ST15 and ST17). **Lys346**, which is an inhibitor in SF6, interacts with the sugar phosphate backbone of the DNA extensively, in addition to making contacts with DNA bases (ST14). **Asn351**, which is an enhancer and inhibitor in Fig. 3, is one of the major amino acids that mediate protein-protein interactions in the C-terminal basic tail (ST14 and ST17). **Asn349** of the recognition helix of one finger, which is an enhancer and inhibitor in Fig. 3, interacts with the second half of the C-terminal basic tail of the other finger, including interaction with residues Lys357, Lys358, Glu359, and Gln362, and causes minor conformational change to the C-terminal. Both of Asn349 and Asn351 of the C-terminal basic tail of both fingers interact with the DNA backbone and minor groove extensively, and form an extended protein-DNA and protein–protein interaction interface; they bind to alpha helix of the DNA all through numerous hydrogen bonds & vdW contacts (ST14 and ST17). **Asn339**, which is an enhancer and inhibitor in Fig. 3, forms a hydrogen bond with Arg339 (protein-protein). It also makes direct hydrogen bonds and van der Waals contacts with DNA bases. For instance, It forms a hydrogen bond with A7′ (protein-DNA) (ST14 and ST16). **Pro353**, which is an enhancer and inhibitor in SF7, is one of the major amino acids that mediates protein-protein interactions in the C-terminal basic tail. This residue interacts with the DNA backbone and minor groove extensively, and forms an extended protein-DNA and protein–protein interaction interface; it binds to alpha helix through numerous hydrogen bonds & vdW contacts (ST14, ST15, and ST17). Similarly, **Leu354**, which is an enhancer and inhibitor in Fig. 7, is one of the major amino acids that mediates protein-protein interactions in the C-terminal basic tail. It interacts with the DNA backbone and minor groove extensively, forms an extended protein-DNA and protein–protein interaction interface, and binds to alpha helix through numerous hydrogen bonds & vdW contacts (ST14, ST15, and ST17). **Leu347**, of the recognition helix of one finger, which is an enhancer and inhibitor in Fig. 7, interacts with the second half of the C-terminal basic tail of the other finger (including interactions with residues Lys357, Lys358, Glu359, and Gln362), causes minor conformational change to the C-terminal, and makes direct hydrogen bonds and van der Waals contacts with DNA bases (ST14 and ST17). **Leu327** and **Leu343**, which are enhancers and inhibitors in Fig. 7, make direct hydrogen bonds and van der Waals contacts with DNA bases. Specifically, Leu327 makes van der Waals contacts with A13 and G14, suggesting that flanking sequences outside the core recognition site (GATA) may affect DNA binding by GATA3 (ST14). **Glu359**, in the second half of the C-terminal basic tail of one finger, is an enhancer in SF2, and interacts with residues at the end of the recognition helix of the other finger, causing minor conformation to the C-terminal. Those interacting residues include: Leu347, His348, Asn349, and Ile350 (ST17). **His348** at the end of the recognition helix of one finger, which is an enhancer and inhibitor in SF4, interacts with the second half of the C-terminal basic tail of the other finger (including residues Lys357, Lys358, Glu359, and Gln362), causing minor conformational change to the C-terminal. In addition, His348 interacts extensively with the DNA backbone through the sugar phosphate and with the minor groove through the DNA bases, hence forming an extended protein-DNA and protein–protein interaction interface (ST14, ST15, and ST17). **Ile350**, at the end of the recognition helix of one finger, which shows to be an enhancer and inhibitor in SF5, interacts with the second half of the C-terminal basic tail of the other finger via residues: Lys357, Lys358, Glu359, and Gln362, thus causing minor conformational change to the C-terminal. In addition, Ile350, of the C-terminal basic tail, interacts with the DNA backbone and minor groove extensively, forming an extended protein-DNA and protein–protein interaction interface; it binds to alpha helix through numerous hydrogen bonds & vdW contacts (ST14, ST15, and ST17). **Ile361**, of the first half of the C-terminal basic tail, which is an enhancer and inhibitor in SF5, contributes to the DNA backbone binding (ST14). **Thr355**, which is an enhancer and inhibitor in Fig. 9, is one of the major amino acids that mediates protein-protein interactions in the C-terminal basic tail. In addition, this residue interacts with the DNA backbone and minor groove extensively, forming an extended protein-DNA and protein–protein interaction interface; it binds to alpha helix through numerous hydrogen bonds & vdW contacts (ST14, ST15, and ST17). **Thr326**, which is an enhancer and inhibitor in Fig. 9, makes a hydrogen bond with Asn339. In addition, Thr326 makes direct hydrogen bonds and van der Waals contacts with DNA bases. For instance, it makes vdW contacts with T8′. It also interacts with the sugar phosphate backbone of the DNA extensively (ST14, ST16). **Gln362,** of the second half of the C-terminal basic tail of one finger, which is an enhancer in Fig. 6, interacts with residues Leu347, His348, Asn349, and Ile350 at the end of the recognition helix of the other finger, and causes minor conformation to the C-terminal (ST17). **Met356**, which is an enhancer and inhibitor in Fig. 8, is one of the major amino acids that mediates protein-protein interactions in the C-terminal basic tail. Among its interacting residues are: Tyr344, Tyr345, His348, and Arg352 in the C-terminal, mainly shaping its conformation. It binds to alpha helix through numerous hydrogen bonds & vdW contacts, and interacts with the DNA backbone and minor groove extensively, forming an extended protein-DNA and protein–protein interaction interface (ST14, ST15, and ST16). **Tyr344**, which is an enhancer and an inhibitor in SF10, is one of the major amino acids that mediates protein-protein interactions in the C-terminal basic tail. Its main interactions are with Tyr345, His348, Arg352, and Met356 in the C-terminal, mainly affecting its conformation. In addition, Tyr344, interacts with the sugar phosphate backbone of the DNA extensively (ST14 and ST17).

**Supplementary Table 14 (ST14). Protein-DNA Interactions from Experiments (OPP Model) [16]**

|  | **DNA Backbone** | **DNA Bases** | **Computation** |
| --- | --- | --- | --- |
| **Ala340** | - interacts with | - interacts with | - Enhancer - Inhibitor |
| **Arg364** | - van der Waals (vdW) with sugar ring | - hydrogen bond (hb) with Thymine 6 (T6’) on strand Y opposite (opp.) to Adenine 17 (A17) on strand X - part of GATA sequence (seq.) - specific to chain B - vdW with neighbor bases | - Inhibitor |
| **Arg329** |  | - hb with Guanine 14 (G14) on strand X-part of GATA seq. - specific for chain B - hb with T8’ on Y strand and it is the amino acid (AA) complement to A15 on X strand - vdW with DNA bases | - Inhibitor |
| **Arg352** |  | - binds to alpha helix via numerous hb & vdW | - Inhibitor |
| **Arg312** | - interacts with | - interacts with | - Inhibitor |
| **Arg330** | - interacts with | - interacts with | - Inhibitor |
| **Lys346** | - interacts with sugar phosphate | - interacts with | - Inhibitor |
| **Asn351** | - interacts with - binds to minor groove | - binds to alpha helix via numerous hb & vdW | - Enhancer - Inhibitor |
| **Asn349** | - interacts with - binds to minor groove | - binds to alpha helix via numerous hb & vdW | - Enhancer - Inhibitor |
| **Asn339** |  | - hb with A7’on strand Y opposite to T16 on strand X - chain B & chain A - vdW with DNA bases | - Enhancer - Inhibitor |
| **Pro353** |  | - binds to alpha helix via numerous hb & vdW | - Enhancer - Inhibitor |
| **Leu354** |  | - binds to alpha helix via numerous hb & vdW | - Enhancer - Inhibitor |
| **Leu347** |  | - hb & vdW with DNA bases | - Enhancer - Inhibitor |
| **Leu327** |  | - hb with DNA bases - vdW with A13 & with G14 - specific to chain B | - Enhancer - Inhibitor |
| **Leu343** |  | - hb & vdW with DNA bases | - Enhancer - Inhibitor |
| **His348** | - interacts with sugar phosphate backbone extensively | - contacts with DNA bases | - Enhancer - Inhibitor |
| **Ile350** |  | - binds to alpha helix via numerous hb & vdW | - Enhancer - Inhibitor |
| **Ile361** | - contributes to backbone binding |  | - Enhancer - Inhibitor |
| **Thr355** |  | - binds to alpha helix via numerous hb & vdW | - Enhancer - Inhibitor |
| **Thr326** | - interacts with sugar phosphate backbone extensively | - hb with DNA bases - specific to chain B - vdW with T8’ on Y strand and it is the AA complement to A15 on X strand | - Enhancer - Inhibitor |
| **Met356** |  | - binds to alpha helix via numerous hb & vdW | - Enhancer - Inhibitor |
| **Tyr344** | - interacts with sugar phosphate backbone extensively |  | - Enhancer - Inhibitor |

**Supplementary Table 15 (ST15). Protein-DNA Interactions from Experiments (ADJ Model) [16]**

|  | **DNA Backbone** | **DNA Bases** | **Computation** |
| --- | --- | --- | --- |
| **Arg352** | Interacts with | Binds to minor groove | - Inhibitor |
| **Lys357** | Interacts with | Binds to minor groove | - Enhancer - Inhibitor |
| **Lys358** | Interacts with | Binds to minor groove | - Enhancer - Inhibitor |
| **Pro353** | Interacts with | Binds to minor groove | - Enhancer - Inhibitor |
| **Leu354** | Interacts with | Binds to minor groove | - Enhancer - Inhibitor |
| **His348** | Interacts with | Binds to minor groove | - Enhancer - Inhibitor |
| **Ile350** | Interacts with | Binds to minor groove | - Enhancer - Inhibitor |
| **Thr355** | Interacts with | Binds to minor groove | - Enhancer - Inhibitor |
| **Met356** | Interacts with | Binds to minor groove extensively | - Enhancer - Inhibitor |

**Supplementary Table 16 (ST16). Protein-Protein Interactions from Experiments (OPP Model) [16]**

|  | **Amino Acids** | **Computation** |
| --- | --- | --- |
| **Asn339** | Hydrogen bond with Arg339 | - Enhancer - Inhibitor |
| **Thr326** | Hydrogen bond with Asn339 | - Enhancer - Inhibitor |
| **Met356** | Tyr344, Tyr345, His348, Arg352 | - Enhancer - Inhibitor |

**Supplementary Table 17 (ST17). Protein-Protein Interactions from Experiments (ADJ Model) [16]**

|  | **Interaction w/ Amino Acids** | **Computation** |
| --- | --- | --- |
| **Arg352** | Tyr344, Tyr345, His348, Met356 | - Inhibitor |
| **Lys357** | Leu347, His348, Asn349, Ile350 | - Enhancer - Inhibitor |
| **Lys358** | Leu347, His348, Asn349, Ile350 | - Enhancer - Inhibitor |
| **Asn351** | Major in Protein-Protein interactions | - Enhancer - Inhibitor |
| **Asn349** | Lys357, Lys358, Glu359, Gln362 | - Enhancer - Inhibitor |
| **Pro353** | Major in Protein-Protein interactions | - Enhancer - Inhibitor |
| **Leu354** | Major in Protein-Protein interactions | - Enhancer - Inhibitor |
| **Leu347** | Lys357, Lys358, Glu359, and Gln362 | - Enhancer - Inhibitor |
| **Glu359** | Leu347, His348, Asn349, Ile350 | - Enhancer |
| **His348** | Lys357, Lys358, Glu359, and Gln362 | - Enhancer - Inhibitor |
| **Ile350** | Lys357, Lys358, Glu359, and Gln362 | - Enhancer - Inhibitor |
| **Thr355** | Major in Protein-Protein interactions | - Enhancer - Inhibitor |
| **Gln362** | Leu347, His348, Asn349, Ile350 | - Enhancer - Inhibitor |
| **Tyr344** | Tyr345, His348, Arg352, Met356 | - Enhancer - Inhibitor |
| **Tyr345** | Tyr344, His348, Arg352, Met356 | - Enhancer - Inhibitor |
